# Qualitative dynamics semantics for SBGN process description

**DOI:** 10.1186/s12918-016-0285-0

**Published:** 2016-06-16

**Authors:** Adrien Rougny, Christine Froidevaux, Laurence Calzone, Loïc Paulevé

**Affiliations:** Laboratoire de Recherche en Informatique UMR CNRS 8623, Université Paris-Sud, Université Paris-Saclay, Orsay Cedex, 91405 France; Institut Curie, PSL Research University, INSERM, U900, Mines Paris Tech, Paris, F-75005 France

**Keywords:** Modeling of dynamics, Reaction networks, SBGN-PD, Qualitative dynamics, Automata networks

## Abstract

**Background:**

Qualitative dynamics semantics provide a coarse-grain modeling of networks dynamics by abstracting away kinetic parameters. They allow to capture general features of systems dynamics, such as attractors or reachability properties, for which scalable analyses exist. The Systems Biology Graphical Notation Process Description language (SBGN-PD) has become a standard to represent reaction networks. However, no qualitative dynamics semantics taking into account all the main features available in SBGN-PD had been proposed so far.

**Results:**

We propose two qualitative dynamics semantics for SBGN-PD reaction networks, namely the general semantics and the stories semantics, that we formalize using asynchronous automata networks. While the general semantics extends standard Boolean semantics of reaction networks by taking into account all the main features of SBGN-PD, the stories semantics allows to model several molecules of a network by a unique variable. The obtained qualitative models can be checked against dynamical properties and therefore validated with respect to biological knowledge. We apply our framework to reason on the qualitative dynamics of a large network (more than 200 nodes) modeling the regulation of the cell cycle by RB/E2F.

**Conclusion:**

The proposed semantics provide a direct formalization of SBGN-PD networks in dynamical qualitative models that can be further analyzed using standard tools for discrete models. The dynamics in stories semantics have a lower dimension than the general one and prune multiple behaviors (which can be considered as spurious) by enforcing the mutual exclusiveness between the activity of different nodes of a same story. Overall, the qualitative semantics for SBGN-PD allow to capture efficiently important dynamical features of reaction network models and can be exploited to further refine them.

**Electronic supplementary material:**

The online version of this article (doi:10.1186/s12918-016-0285-0) contains supplementary material, which is available to authorized users.

## Background

A full understanding of a biological process requires its investigation from two points of view: a functional point of view, and a mechanistic point of view. From the functional point of view, discovering the structures and the functions taking part in the biological process is of crucial importance, while from the mechanistic point of view, the focus is on deciphering the mechanisms underlying these functions.

Cellular processes are mostly studied at the molecular scale. In that case, describing a cellular process from the functional point of view consists in describing the molecular activities that underpin it, as well as the influences these activities have on each other. Such descriptions are generally represented in the form of *influence graphs*. Describing cellular processes from the mechanistic point of view involves describing the molecular entities and the molecular processes that take part in the cellular process. These descriptions are mainly represented in the form of *reaction networks*. In reaction networks, nodes represent molecular entities (e.g. a molecule, a complex, an ion) and arcs represent reactions or influences of some molecular entities on reactions. Reaction networks allow to model a large variety of biological processes, such as metabolic [1] or signaling processes [2]. The majority of available comprehensive reaction networks model metabolic processes (see [3] for an example of a comprehensive metabolic network). Yet, comprehensive networks modeling signaling processes with several hundreds of nodes have been built during this last decade [4–6].

Standardized representations of molecular networks (and in particular reaction networks) have arose with the continuously growing available biological knowledge. One of the main standards is the Systems Biology Graphical Notation (SBGN) [7].

Molecular networks such as influence graphs and reaction networks are static representations. One of the main motivations for establishing dynamical semantics on a static map is the ability to verify if the knowledge gathered by the map is sufficient to reproduce known behaviors. Indeed, analyzing the dynamics of the cellular processes they describe requires building formal dynamical models that can then be either analyzed exhaustively or *automatically* checked against dynamical properties of interest (referred to as *model-checking*). Influence graphs are conveniently interpreted using qualitative semantics (e.g. automata networks [8], Boolean networks [9]) whereas reaction networks are usually interpreted using quantitative semantics (e.g. Ordinary Differential Equations (ODEs)).

In this paper, we are interested in qualitative semantics for modeling reaction networks expressed in the Systems Biology Graphical Notation Process Description Language (SBGN-PD) using asynchronous automata networks. In the rest of this section, we first present SBGN-PD. We then give an overview of the standard techniques usually used to model reaction networks, before presenting the asynchronous automata network formalism. Finally we motivate the two qualitative semantics introduced in this article.

### SBGN process description

SBGN consists of three complementary languages: Process Description (SBGN-PD), Activity Flow (SBGN-AF) and Entity Relationship (SBGN-ER). Each of these languages allows us to represent biological knowledge at a different level of abstraction: SBGN-PD at the reaction level, SBGN-AF at the more abstract activity level and SBGN-ER at the conceptual influence level. These languages rely on the Systems Biology Ontology (SBO) [10]: each glyph of the three languages is associated to a term from SBO. Therefore, SBGN is more than a standard way to represent reaction networks. It also allows to standardize the concepts and vocabulary used to model biological processes. As we are interested in modeling reaction networks, we focus on SBGN-PD in this paper.

SBGN-PD has four main classes of glyphs, that form together the nodes and arcs of any SBGN-PD map:

- *Entity Pool Nodes (EPN)*: An EPN represents a pool of molecular entities, a *perturbing agent*, a *source* or a *sink*. Source nodes (*resp.* sinks nodes) are used when one does not want to specify the molecular entities from (*resp.* into) which a particular EPN is synthetised (*resp.* degraded). There are four subtypes of EPNs: *unspecified entity*, *simple chemical*, *macromolecule* and *nucleic acid feature*.
- *Process Nodes (PN) and Flux Arcs*: A PN represents a molecular process. Flux arcs, that link EPNs to PNs, represent consumption and production of EPNs by processes. There are six subtypes of processes: *process*, *omitted process*, *uncertain process*, *association*, *dissociation*, and *phenotype*.
- *Modulation Arcs*: Modulation arcs, that link EPNs to PNs, represent the possible effects EPNs have on processes. There are five subtypes of modulations: *modulation*, *stimulation*, *catalysis*, *inhibition* and *necessary stimulation*.
- *Logical Operators and Logic Arcs*: The AND operator represents necessary conditions for modulations to be performed, the OR operator sufficient conditions for modulations to be performed, and the NOT operator the non-existence of a modulation. Logic arcs link EPNs to logical operators, or logical operators to other logical operators.

SBGN-PD contains five additional types of glyphs: *compartments*, *clone markers*, *reference nodes*, *equivalence arcs* and *submaps*. The compartment glyph is used to represent compartments, whereas the other four glyphs are used to refer to other nodes already present in the map. Each of these glyphs will not be interpreted *per se* in the semantics presented in the next section as they do not have any meaning when considering the dynamics of the network. However, the location of an EPN into a specific compartment is taken into account: two EPNs that share exactly the same attributes but are in different compartments are considered as different EPNs. Then, since we focus on qualitative semantics, we do not consider the stoichiometry of processes. Also, the semantics of the NOT operator given in the specification has no meaning regarding dynamics of networks: hence, we will not take into account this operator. Finally, reversible processes are not taken into account as their representation (and therefore their detection) is based on the spatial localisation of their reactants/products. However, a reversible process can be taken into account by rewriting it into two processes (one forward and one backward process) in the map.

**Fig. 1:**
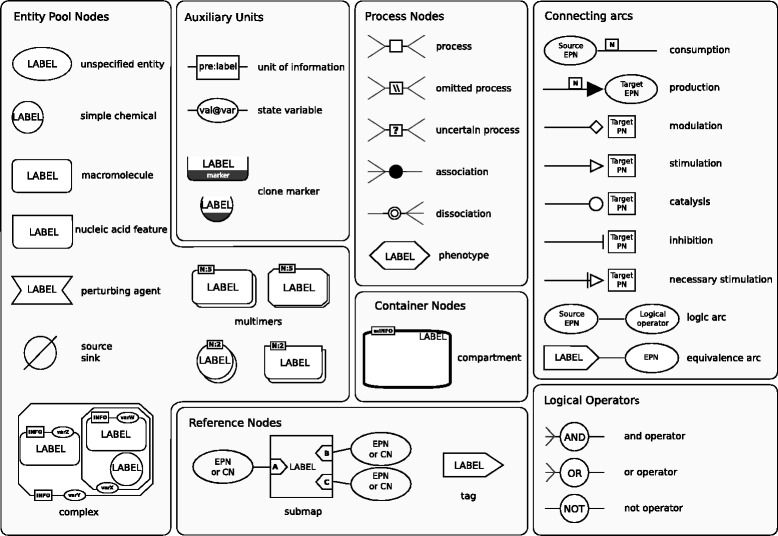
Reference card of the SBGN-PD language from [7]. Every glyph of SBGN-PD is associated to the biological concept it represents

The correspondence between the different glyphs of SBGN-PD and the biological concepts they represent is given in Fig. 1. Real-life examples of SBGN-PD maps are given in Figs. 5 and 9. SBGN maps can be stored and exchanged in the SBGN-ML format [11] and edited by a variety of software (e.g. VANTED’s add-on SBGN-ED [12], CellDesigner [13]).

In the rest of the article, we will refer to an EPN linked to a PN by a consumption arc (resp. production arc, modulation arc, stimulation arc, catalysis arc, inhibition arc and necessary stimulation arc) as a *reactant* (resp. *product*, *modulator*, *stimulator*, *catalyzer*, *inhibitor* and *necessary stimulator*) of the process represented by the PN.

For the sake of simplicity, we will use the same terms for the glyphs and arcs of SBGN-PD and the concepts they represent. For example, we use the term “EPN” to refer to the node just as well as to the entity pool it represents; the term “stimulation” refers to a stimulation arc and to the stimulation it represents. Also, terms “EPN”, “process” and “modulation” refer to the associated concept (or glyph) as well as to all concepts that are subtypes of these concepts. For example, the term “modulation” also refers to a stimulation, and the term “process” also refers to a phenotype.

### Qualitative dynamics of reaction networks

The dynamics of reaction networks is usually modeled with quantitative semantics such as population (stochastic) semantics [14–19], or continuous deterministic semantics (ODEs) [18–22]. These models rely on multiple parameters, including reaction kinetics, that are often difficult to measure, thus limiting their applicability.

Formalisms that do not rely on kinetic parameters, such as Flux Balance Analysis [23], are also widely used to model reaction networks. However, these formalisms are based on the steady-state assumption and do not allow to model the dynamics of networks.

We can find in [24] a classification of the main modeling formalisms for reaction networks (and in particular metabolic networks) depending on whether they lead to quantitative or qualitative models. In this study, authors also propose a unified framework to integrate these different formalisms by means of graph transformations.

In addition, let us mention qualitative formalisms such as Boolean or discrete networks, that are used to model the dynamics of molecular networks and do not consider any kinetic parameters. This type of modeling introduces a notion of *threshold* for the number of molecules (population) of the modeled chemical species. To each chemical species is assigned a number of thresholds and the population of each species is quantized following its thresholds. Species are then modeled by variables with finite domains, and the changes in the values of the different variables are no longer considered as continuous phenomena but discrete transitions.

Qualitative modeling has primarily been introduced by S. Kauffman in order to model the dynamics of gene regulatory networks, and are now also used to model the dynamics of other types of networks, such as signaling networks. Several formalisms have been proposed in that respect, depending on the type and the size of the domains considered for the variables: Boolean networks [9, 25], multi-valued models [26, 27], bounded Petri nets [28] or fuzzy logic [29]. The dynamics of qualitative models is coarser than the one of the quantitative models, but it helps the tractability of the analysis of attractors, that are the final states of the system, and reachability properties while abstracting away kinetic parameters. On medium-size models, the computation of the exhaustive dynamics is possible, whereas methods to handle large-size qualitative models are emerging [30–32].

Qualitative formalisms have also been applied to model the dynamics of reaction networks where, in addition to influences, consumption and production of molecules are taken into account. The main contribution to this field is the Biological Abstract Machine (BIOCHAM) modeling environment [19], that allows to analyze reaction networks using a Boolean semantics, and that we present hereafter.

### Boolean semantics of BIOCHAM

In the BIOCHAM Boolean semantics [19] each molecular entity of the network can be either absent or present. Each compound is associated to a Boolean variable whose binary value represents its state (*false* or 0 for absent and *true* or 1 for present). In BIOCHAM, a reaction *A*+*B*→*C*+*D* is interpreted by four different Boolean transitions (where ∧ denotes the AND logical operator):

- *A*∧*B*→*A*∧*B*∧*C*∧*D*
- *A*∧*B*→¬*A*∧*B*∧*C*∧*D*
- *A*∧*B*→*A*∧¬*B*∧*C*∧*D*
- *A*∧*B*→¬*A*∧¬*B*∧*C*∧*D*

Occurrences of variables *A* and *B* in the left-hand side of the transition express the fact that all reactants must be present for the reaction to occur; occurrences of *C* and *D* in the right-hand side express the fact that the occurrence of the reaction causes the presence of all the products; finally the combination of variables *A* and *B* or of their negation in the right-hand side expresses the fact that reactants may or may not be completely consumed by the reaction.

This semantics can take into account stimulation (and in particular catalysis) by adding the stimulator to the reaction as both a reactant and a product. The corresponding transitions can then be fired only if the stimulator is present, and this stimulator remains present as it appears among the products of the reaction.

The Boolean semantics of BIOCHAM is an over-approximation of the quantitative population semantics [33], in the sense that every trace of the quantitative semantics has a corresponding trace in the Boolean semantics. Hence the absence of a behavior in the Boolean semantics guarantees the absence of this behavior in the population semantics.

### Asynchronous automata networks

An *automata network* (AN) is defined as a set of finite-state automata, where each automaton has a finite set of exclusive states called *local states*. At any time, each automaton has one and only one local state active, and the *global state* of an AN is the set of the active local states of its automata. Transitions between local states of each automaton are conditioned by the local state of other automata in the network.

More formally, an AN is defined as a triple (*Σ*,*S*,*T*) where

- *Σ* is a finite set of automaton names;
- For all *a*∈*Σ*, *S*(*a*)={*a*_*i*_,⋯,*a*_*j*_} is the finite set of local states of automaton *a*. We note $S = \prod _{a\in \Sigma }S(a)$ the set of all the *global states* of the AN.
- $T \subseteq \left \{{a}_{i}\overset {\ell }{\rightarrow }{a}_{j} \mid a\in \Sigma, a_{i}\in S(a), a_{j} \in S (a), \ell \subset \right.$$\left.\bigcup _{b\in \Sigma, b\neq a} S(b) \right \}$ is the finite set of local transitions with conditions (*ℓ*).

**Fig. 2:**
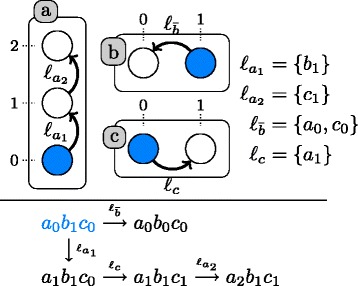
An example of asynchronous automata network and its transition graph. *Top:* an asynchronous automata network composed of the 3 automata *a*, *b* and *c*. Automata are represented by labeled boxes, and their local states by circles identified with the ticks. For instance, the circle ticked 1 in the automaton *a* is the state 1 of *a*, noted *a*_1_. Local transitions are represented by directed labeled edges, where the labels indicate the set of conditions that have to be satisfied for firing the transition. The local states in blue represent a potential global state of the automata network: the state *a*_0_,*b*_1_,*c*_0_. *Bottom:* the transition graph of the asynchronous automata network, from the global initial state represented in blue. This graph represents all transitions that can be successively fired from the global initial state. For example, from the global initial state, it is possible to fire the transition labeled $l_{a_{1}}$ or the transition labeled $l_{\overline {b}}$. One of these two will be fired non-deterministically. Firing transition $l_{a_{1}}$ will change the state of *a* from 0 to 1, hence replacing *a*_0_ with *a*_1_ in the global state of the network, becoming *a*_1_,*b*_1_,*c*_0_. Firing transition $l_{\overline {b}}$ will change the state of *b* from 1 to 0, hence replacing *b*_1_ with *b*_0_ in the global state of the network, becoming 〈*a*_0_,*b*_0_,*c*_0_〉

Figure 2 gives an example of AN. This AN is defined by the triple (*Σ*,*S*,*T*) as follows:

$$\begin{aligned} \Sigma &= \{a, b, c\}\\ S(a) &= \{ a_{0}, a_{1}, a_{2}\} \\ S(b) &= \{ b_{0}, b_{1}\} \\ S(c) &= \{ c_{0}, c_{1}\} \end{aligned} $$

$$\begin{aligned} T &= \left\{ a_{0} \xrightarrow {\{b_{1}\}} a_{1}, a_{1} \xrightarrow {\{c_{1}\}} a_{2}, \right.\\&\qquad \left. b_{1} \xrightarrow {\{a_{0},c_{0}\}} b_{0},c_{0} \xrightarrow {\{a_{1}\}} c_{1} \right\} \end{aligned} $$

Given a (global) state *s*∈*S* of an AN, there is a transition to a state *s*^′^∈*Siff* there exists a local transition $a_{i}\overset {\ell }{\rightarrow } a_{j} \in T$ such that the automaton *a* is at state *a*_*i*_ in *s*, and all local states in *ℓ* are present in *s*. The state *s*^′^ is then the state *s* where the local state *a*_*i*_ of automaton *a* has been replaced with *a*_*j*_. Such dynamics are called *asynchronous* as one and only one local transition is applied at a time. Note that from a state *s*, there may exist several applicable local transitions leading to non-deterministic dynamics.

More formally, given an AN (*Σ*,*S*,*T*), the global asynchronous transition relation → included in *S*×*S* is defined by:

$$\begin{array}{*{20}l} s \rightarrow s' \stackrel{\Delta}\Leftrightarrow \exists &{a}_{i}\overset{\ell}{\rightarrow}{a}_{j}\in T: a_{i}\in s, \ell\subset s, a_{j}\in s' \\ & \forall c_{k}\in s: c_{k}\neq a_{i} \Rightarrow c_{k}\in s' \end{array} $$

In the scope of the article, we consider only the asynchronous update scheme for ANs, widely integrated in software. Other update schemes can be of interest in the scope of reaction networks, in particular the general update scheme which mixes asynchronous and synchronous automata transitions.

Figure 2 shows an asynchronous AN and all the transitions that can be applied from a global initial state, resulting in a so-called state transition graph.

#### Relation of automata networks with petri nets

Asynchronous ANs are very close to so-called *1-bounded* Petri nets [34] (at most one token per place): one can encode an AN into Petri net with one place per local state of the automata, one transition per local transition, having one incoming, one outgoing arc and any number of read-arcs, and where places have at most one token [35]. Therefore, all semantics formalized with ANs, and in particular the semantics we propose in the following sections, can be encoded with Petri nets. An example of such an encoding for the AN of Fig. 2 is given in Additional file 1 with an illustration of the differences between the two approaches.

The stories semantics we introduce merges sets of SBGN-PD entities into *components*: a component aims at representing a molecular entity whose current state corresponds to one of the EPNs composing it. Therefore, we distinguish three features in our models: the components, their local states, and the transitions (processes). 1-bounded Petri nets have only two features, places (for local states/EPNs) and transitions, and therefore cannot represent explicitly components. Only computations on their structure and dynamics allow to uncover mutual exclusive places, delimiting components. On the other hand, ANs directly offer the adequate model structure: automata (components), local states (EPNs), and transitions.

Moreover, in order to address large SBGN-PD maps in the Application to the *RB/E2F* map section, we rely on scalable computational techniques that are currently defined only for ANs, as they exploit the explicit modeling in automata.

More elaborated encodings in general Petri nets of qualitative models such as multi-valued networks have been proposed [28], but they cannot be used straightforwardly for the general ANs we consider here: local states of automata are not necessarily ordered, i.e., there can be local transitions between any local states of each automaton (e.g., one can change from *a*_1_ to *a*_3_ without having to go through *a*_2_). Petri nets extensions such as colored Petri nets [36] could provide an alternative encoding, but for the sake of notation simplicity, the AN formalism has been preferred in this paper.

### Motivation

So far, no qualitative semantics taking into account the main features of SBGN-PD has been proposed. To remedy it, we introduce two qualitative semantics, namely the *general semantics* and the *stories semantics*, that both take into account the main features of SBGN-PD. These two semantics are formalized using asynchronous automata networks, that is a simple yet expressive formalism to formalize dynamical systems.

While the general semantics extends BIOCHAM’s Boolean semantics by taking into account the main features of SBGN-PD, the stories semantics proposes a different interpretation of reaction networks. The stories semantics allows to focus on physical states (e.g. unphosphorylated/phosphorylated) of molecular entities rather than on the entities themselves. Applied to a reaction network, this semantics collapses the different physical states of a given molecular entity into a unique abstract entity, called *story*. This leads to models that are more understandable and closer to the way experts apprehend biological processes, while still considering all the detailed mechanisms depicted in reaction networks. In addition, by lumping several entities in so-called stories, the stories semantics reduces the dimension of the dynamics (number of variables and number of states), which may increase the scalability of its analysis.

The rest of this paper is organized as follows. In the ‘Results’ section, we define the general semantics and the stories semantics, and illustrate them with a large-scale map of the cell cycle centered on the RB/E2F dynamics (*RB/E2F* map for short). In the ‘Discussion’ section we compare stories to related work, and discuss the applicability of the stories semantics to various types of reaction networks, as well as the application of model-checking to the resulting dynamical models. Finally, the ‘Methods’ section gives the formal definitions and encodings in asynchronous automata networks of our qualitative semantics.

## Results

In this section, we propose two different qualitative dynamics semantics for SBGN-PD networks expressed in the asynchronous automata network framework. First we propose a *general semantics* that takes into account all the main features of SBGN-PD maps. Then we introduce a completely new qualitative dynamics semantics that we call the *stories semantics*. Finally, we illustrate both semantics on a cell cycle detailed map.

### General semantics

In the general semantics, we consider that an EPN can be either present or absent in the system. Therefore, in this context, we choose to interpret EPNs by Boolean values rather than by bounded-integers as we do not have any a priori information on differential effects EPNs may have based on (relative) quantities. Analogously to EPNs, a process can be either occurring or non-occurring, and a modulation either active or inactive. Occurrence of a process (i.e., its transition from a non-occurring to an occurring state) is conditioned by the presence of all its reactants, and the activity of all its modulations.

The general semantics extends the Boolean semantics of BIOCHAM by taking into account inhibitions as well as the AND and OR logical operators.

#### Dealing with modulations

The input of a modulation can either be a single EPN or a set of EPNs structured by a logical function (represented in SBGN-PD by logical operators and arcs). A modulation is said to be active if its input is satisfied, and inactive otherwise. If the input is a single EPN, satisfaction of the input means that the EPN must be present; if the input is a logical function, satisfaction of the input means that the states of the modulators that form the function must satisfy it.

A single process may be targeted by more than one modulation. In this particular case the mechanism underlying the global modulation of the process is unknown (or not specified), otherwise it would be structured by some logical function. Hence, for the dynamics to be as general as possible while taking into account the effects of modulations, we choose to consider that a process can change from a non-occurring to an occurring state only if the following two conditions are satisfied:

- all its necessary stimulations are active and
- at least one of its stimulations (including catalyses) is active or at least one of its inhibition is inactive.

With this interpretation, a process modulated by both a stimulator and an inhibitor can occur if its stimulator and its inhibitor are both present, both absent, or if its inhibitor is absent and its stimulator present.

This weak constraint (but meaningful in terms of biology) ensures that the obtained dynamics includes all dynamics that would be obtained with more restrictive conditions, and in particular the one that would be obtained from the model built with the (unknown) accurate logical functions. Therefore, if a process can get activated with a stronger constraint (for example: all inhibitions must be inactive), it can get activated considering our weak constraint. Note that we do not take into account modulations that are neither stimulations nor inhibitions, as we do not know their effect on the processes.

#### From SBGN-PD to automata networks under the general semantics

The semantics we propose in this paper are expressed in terms of asynchronous automata networks (AN). Recall that asynchronous automata networks gather a set of automata with a certain number of local states, and a set of local state transitions within each automaton that can be constrained with conditions on the active local states of other automata in the network.

In the scope of the general semantics of SBGN-PD maps, each EPN is associated to one automaton having two local states labeled 0 (for absent) and 1 (for present). Similarly to EPNs, each process is associated to one automaton having two local states labeled 0 (for non-occurring) and 1 (for occurring).

Local state transitions are built as follows:

- a process can change from its non-occurring to its occurring state *iff* all its reactants (that are not source EPNs) are present, all its necessary stimulations are active, and at least one of its stimulations is active or one of its inhibition is inactive;
- a process can change from its occurring to its non-occurring state *iff* all its products (that are not sink EPNs) are present;
- an EPN can change from an absent to a present state *iff* there is an occurring process that produces it;
- finally an EPN can change from a present to an absent state *iff* there is an occurring process that consumes it and all the products of this process are present.

Note that as we do not have a priori information on the equilibrium of the different processes of the map we model, full consumption of reactants by an occurring process is not made mandatory, exactly as in BIOCHAM’s Boolean semantics. This is achieved by encoding transitions as presented above, and by considering asynchrony: the transition that consumes an EPN may or may not be triggered before the process becomes non-occurring.

**Fig. 3:**
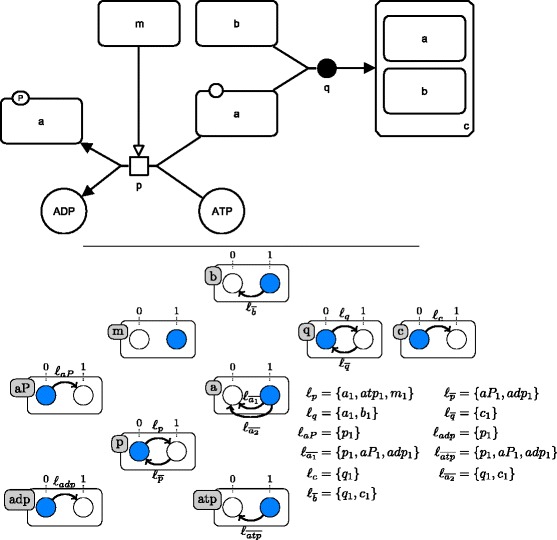
A SBGN-PD process modeled by an asynchronous automata network under the general semantics. *Top:* An example of SBGN-PD map. The legend of the map is given by the SBGN-PD reference card reproduced in Fig. 1. *Bottom:* the asynchronous automata network modeling the SBGN-PD map under the general semantics, with the different automata and for each transition, its firing conditions. The global initial state <*a*_1_,*a* *t* *p*_1_,*b*_1_,*m*_1_,*a* *P*_0_,*a* *d* *p*_0_,*c*_0_,*p*_0_,*q*_0_> is represented in *blue*

Figure 3 shows how a unique process can be modeled by an AN under the general semantics. The full formalization of the general semantics for SBGN-PD maps by asynchronous automata networks is given in the ‘Methods’ section.

An *initial state* defined on a map is a set of EPNs of that map that are considered as being present at time *t*_0_. An initial state of a map can be straightforwardly encoded into a global initial state of an AN built under the general semantics: for each EPN of the initial state of the map, we add the present state of that EPN to the global initial state of the AN.

The exhaustive dynamics of an AN is obtained by computing all transitions from a given global initial state of the model. It results in a finite graph called a *state transition graph* whose nodes are the global states of the AN. This graph may contain cycles indicating oscillations and a node may have several successors, indicating a non-deterministic choice between two transitions.

### Stories semantics

SBGN-PD has been designed in order to model, among others, changes of physical states or locations of molecular entities. For instance, an unphosphorylated protein and its phosphorylated form are two states of the same protein that are represented in SBGN-PD by two different EPNs, and linked together by a process that changes one EPN into the other. Similarly, a molecule involved in an association process can have a free state and a bound state, and a molecular entity involved in a translocation process can have two states, one for each compartment involved. A single molecular entity can be the target of several of those changes, and therefore have several different states, each represented by a different EPN.

A particular state of a molecular entity might correspond to an *active state* of the molecular entity, meaning a state where the entity performs a function. For example, in signaling, a kinase often performs its function only once it gets phosphorylated. Such a kinase activity (for a given molecular entity) will be represented by one kinase activity node in an influence graph, and modeled by one variable that can take two values under a Boolean semantics: 0 (off) when the activity is not performed and 1 (on) when the activity is performed. Hence, within this setting, a kinase will be either active or inactive, but not both at the same time. We say that both states (active and inactive) are *mutually exclusive*. Since, in our example, the active state of the kinase corresponds to its phosphorylated state and the inactive state to its unphosphorylated state, this way of modeling implies that physical states of the kinase are also made mutually exclusive.

The *stories semantics* aims at modeling changes of state of a molecular entity from this perspective. It constrains the general semantics by ensuring that all EPNs representing different states of the same molecular entity are *mutually exclusive*, meaning that they will never be present at the same time.

#### Stories

Given a molecular entity, we define a *story* as a set of EPNs (different from a sink EPN), each representing a different physical state of that molecular entity. Given an SBGN-PD map, a story must respect the following constraints:

- for any two distinct EPNs of the story, there exists a path in the map between the two EPNs such that all the edges of the path are flux arcs and all the EPNs of the path belong to the story;
- if an EPN of the story is a product of a process, then at least one reactant (that can be a source EPN) of that process belongs to the story;
- for two EPNs of the story, there exists no process that consumes both of them;
- for two EPNs of the story, there exists no process that produces both of them.

Constraint (i) considered together with constraints (iii-iv) ensures that all EPNs of a story represent the different states of a given molecular entity that appear by transformation of that entity. Constraints (ii-iv) allow to define a semantics where the EPNs of a story are mutually exclusive: Constraint (ii) ensures that no process can produce an EPN belonging to a story without first consuming an EPN of that story. Constraint (iii) ensures that, for a given process, all its reactants can be present at the same time, so that it can be triggered. Finally, constraint (iv) ensures that a given process can produce all its products.

Two EPNs that are different states of the same molecular entity often share a common SBGN-PD label, that names the molecular entity. Hence an optional constraint (v) allows to ensure that all EPNs of a story represent different states of the same molecular entity:

- all EPNs of the story have the same (SBGN-PD) label (whether it is the label of the EPN itself, or of an element of the EPN in the case where the EPN is a complex).

**Fig. 4:**
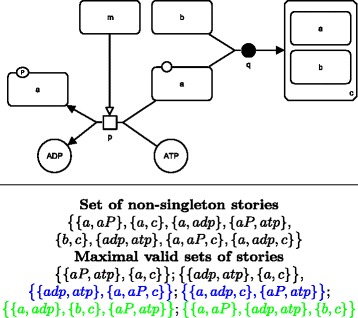
Stories of an SBGN-PD map. *Top:* The SBGN-PD map of Fig. 3. *Bottom:* the set of possible (non-singleton) stories respecting constraints (i-iv) and the maximally valid sets of stories. Final sets are colored in blue, and epn-maximal sets are colored in green. Each of the constants *a*, *b*, *c*, *adp* and *atp* denotes the EPN whose label equals that constant. Constant *aP* denotes the phosphorylated macromolecule labeled “a”

Figure 4 shows an SBGN-PD map together with all its stories containing two or more EPNs, computed with constraints (i-iv).

**Fig. 5:**
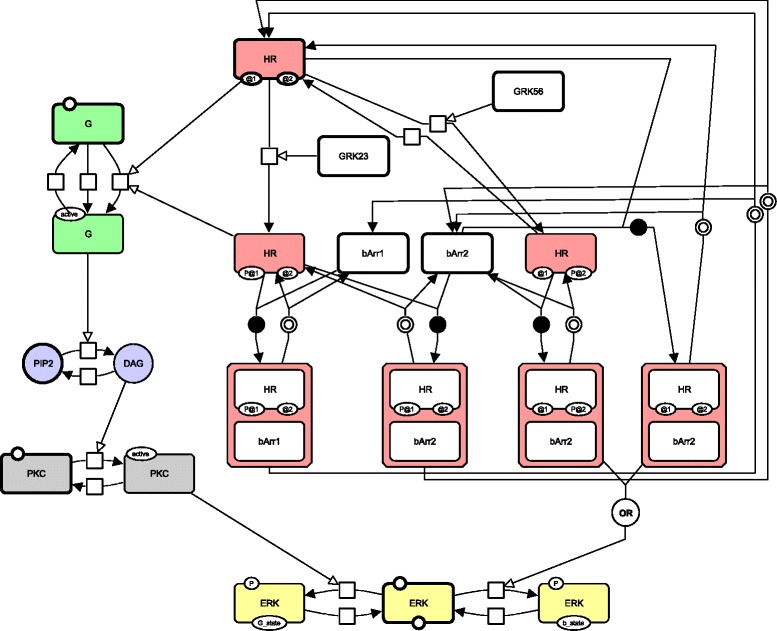
AT _1*A*_R-mediated ERK activation map. This map represents the two main pathways responsible for the AT _1*A*_R-mediated (and more generally 7TMRs receptors-mediated) ERK activation. The AT _1*A*_R receptor activates the (classical) G protein pathway to reach ERK but also the less known *β*-arrestins pathway. These pathways are tightly regulated by the presence of molecules called the G-protein coupled receptor kinases (GRK2/3 and GRK5/6), which act directly on the phosphorylation of the receptor. This map is represented using the SBGN-PD language. EPNs with bold borders constitute the initial state of the map. Every colored EPN belongs to a story, and each color is assigned to a different story. The story in pink focuses on the receptor HR and comprises seven different physical states of this receptor: unbound, phosphorylated (on either of two sites), bound to *β*-arrestin 1 or *β*-arrestin 2. The other stories focus on ERK (*in yellow*), on protein G (*green*), on PIP2 (*blue*), and on PKC (*gray*)

It is worth noticing that, despite the above constraints, the EPNs of a story are not necessarily mutually exclusive in the general semantics. Stories are not emerging properties from the net, and as such, are different from usual structural properties of Petri nets (e.g., siphons, traps, places/transitions invariants; see [37] for a comprehensive survey) which reflect specific dynamical properties of the system. Since the EPNs of a stories are enforced to be mutually exclusive in the stories semantics, they form places invariants (the number of active places is a constant) in this semantics: it is a property of the stories semantics but not a property of the initial map.

An SBGN-PD map may focus on several molecular entities of interest and thus contain several stories. We are therefore interested in characterizing combinations of stories. Since the EPNs of a story are intended to be mutually exclusive, two stories cannot share a same EPN as it would exist in both stories independently. We define a set of stories as *valid* if its stories do not intersect pairwise. Given a map, we are interested in finding one meaningful valid set of stories in order to model that map under the stories semantics. The requirement for a set of stories to be valid might induce a necessary choice between two alternative stories sharing the same EPN. In particular, association processes might lead to alternative stories, one for each compound of the resulting complex, that share the same EPN (the complex). Figure 4 gives all maximally valid sets of stories of the SBGN-PD map introduced previously.

Although computing individual stories is scalable with the map size, the number of valid combinations of stories can be very large, as it depends on both the number of EPNs and the number of individual stories of the map.

In order to drastically reduce the number of candidate valid sets, we define two progressive maximality constraints. (1) A set of stories *S* is said to be *finaliff* (i) it is valid and (ii) there exists no valid set of stories *S*^′^≠*S* such that for every story of *S*, there exists a story of *S*^′^ that is a superset of that story. Note that all final sets are also maximally (in the sense of inclusion) valid. (2) A set of stories *S* is said to be *epn-maximaliff* (i) it is valid and (ii) there exists no valid set of stories *S*^′^≠*S* such that the total number of EPNs in *S*^′^ is greater than the total number of EPNs in *S*. Note that all final sets of stories are maximally valid, and that all epn-maximal sets of stories are final. Figure 4 shows final sets of stories in blue, and epn-maximal sets of stories in green.

Furthermore, additional constraints can be specified following expert knowledge. This requires to focus on particular molecular entities relevant for the biological application.

Finally, in order to apply the stories semantics, the choice of the valid set of stories should be guided by expert knowledge and the specific biological question.

##### Illustration on the *AT *_1*A*_*R-mediated ERK activation* map

Figure 5 shows the *AT*_1*A*_*R-mediated ERK activation* map that was introduced in [22]. It represents the two main pathways responsible for the AT _1*A*_R-mediated (and more generally 7TMRs receptors-mediated) ERK activation. The AT _1*A*_R receptor activates the (classical) G protein pathway to reach ERK but also the less known *β*-arrestins pathway. These pathways are tightly regulated by the presence of molecules called G-protein coupled receptor kinases (GRK2/3 and GRK5/6), which act directly on the phosphorylation of the receptor.

In order to illustrate the concept of stories and of valid sets, we computed all final sets of stories considering constraints (i-iv). There were only two final sets of stories: one including one story for each *β*-arrestin, and one including a story focusing on the receptor. This illustrate the necessary choice between alternative stories induced by the property of validity: as *β*-arrestins can associate with the receptor, one should choose between a story focusing on the receptor and stories focusing on the *β*-arrestins. Stories focusing on the EPNs of the rest of the map were the same in the two sets, namely: a story for protein G, one for PIP2/DAG, one for PKC, and one for ERK.

The set containing a story focusing on the receptor is represented in Fig. 5. This set includes a story that contains all EPNs of the map related to the receptor (i.e. that contains the label "HR"), each representing a particular state of the receptor: unbound, phosphorylated (on either of two sites), bound to *β*-arrestin 1 or *β*-arrestin 2. Hence such a story allows to model the succession of physical states of the receptor, and some of these physical states are also active states: for example, the free receptor can activate protein G when phosphorylated on its first site, and it loses this capacity when associated to any of the *β*-arrestins.

Note that the story containing PIP2 and DAG, represented in blue in Fig. 5, does not respect constraint (v) while it has a biological meaning: this constraint is too stringent for processes that transform small molecules that always have different labels (unlike proteins, for example).

#### From SBGN-PD to automata networks under the *stories semantics*

The stories semantics differs from the general one only in the modeling of EPNs that belong to stories. Instead of modeling each of those EPNs by dedicated automata, a single automaton is declared for each story with one local state per non-source and non-sink EPN of the story. Each automaton of a story also possesses a special local state, referred to as the *empty* state. Each local state of the automaton associated to a story but the empty state corresponds to a physical state of the molecular entity related by the story. As for the empty state, it corresponds to the absence of this molecular entity.

Local state transitions for stories are built as follows:

- a story can change from a (possibly empty) local state to another (not empty) local state *iff* an occurring process consumes the EPN to which corresponds the first local state and produces the EPN to which corresponds the second local state;
- a story can change from a local state to the empty local state *iff* an occurring process consumes an EPN to which corresponds the local state and does not produce any EPN belonging to that story.

Since processes of an SBGN-PD map consume and produce EPNs, and because the semantics of processes and modulations in the general semantics is built upon the presence and absence of EPNs, the notions of presence and absence for EPNs of a story is defined as follows: an EPN of a story is *present* if the automaton associated to the story containing that EPN is in the state corresponding to that EPN; this EPN is *absent* otherwise.

In order to avoid conflicts between processes acting on a same story, we impose the exclusivity between the occurrence of such processes. Therefore, a process acting on a story can occur only if no other process acting on the same story is occurring.

**Fig. 6:**
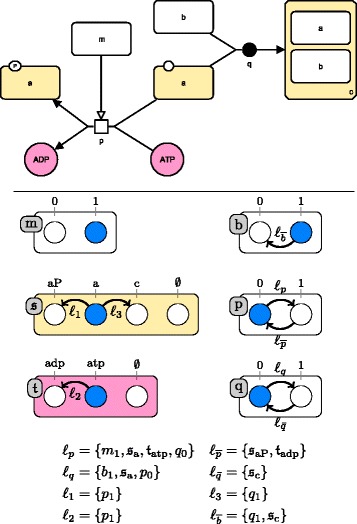
A SBGN-PD map modeled by an asynchronous automata network under the stories semantics. *Top:* the SBGN-PD map from Fig. 3. We chose a final set of two stories $\mathfrak s$ and $\mathfrak t$, whose EPNs are colored in yellow or blue, respectively. *Bottom:* The corresponding asynchronous automata network using the stories semantics with the stories $\mathfrak {s}=\{a,aP,c\}$ and $\mathfrak t=\{adp,atp\}$. Each of the constants *a*,*c*,*a* *d* *p* and *atp* denotes the EPN whose label equals that constant. Constant *aP* denotes the phosphorylated *macromolecule* labeled “a”. The global initial state $\left <\mathfrak {s}_{a},\mathfrak {t}_{atp},b_{1},m_{1},p_{0},q_{0}\right >$ is represented in blue. Note that here, *p* *#* *q*. Therefore *q*_0_∈*l*_*p*_ and *p*_0_∈*l*_*q*_

Figure 6 shows how a simple SBGN-PD map is modelled under the stories semantics. The complete formalization of the stories semantics for SBGN-PD maps into automata networks is provided in the ‘Methods’ section.

Given a map and a set of stories, an initial state of that map must respect the following constraint: two EPNs that belong to the same story cannot be both in the initial state. This constraint is needed so that the initial state does not contradict the property of mutual exclusivity of the EPNs belonging to stories. An initial state of a map can be straightforwardly encoded into a global initial state of an asynchronous AN modeling that map under the stories semantics. All EPNs of the initial state that do not belong to a story are encoded the same way as for the general semantics. For each EPN of the initial state that belongs to a story, we add to the global initial state of the asynchronous AN the local state of the automaton associated to the story that corresponds to that EPN.

#### Relation between the general and the stories semantics

The stories semantics offers a more constrained dynamics than the general semantics, notably by enforcing the mutual exclusiveness of EPNs within a story. In particular, the stories semantics forces the total consumption of the reactant EPNs within stories: considering a process triggering a transformation *A*→*A*^′^, the general semantics allows to produce *A*^′^ while keeping *A* present (its degradation is optional), whereas the stories semantics replaces in one step the activity of *A* with *A*^′^. Intuitively, it results that the stories semantics produces a sub-dynamics of the general semantics.

To each global state *x* in the stories semantics corresponds one global state [x ]in the general semantics where each EPN embedded in a story is in a present state if and only if it is the current local state of its associated story. The two semantics satisfy the following relationships:

##### **Property1**.

Let *x*,*x*^′^ be states where no process is active. If *x*^′^ is reachable from *x* in the stories semantics, then *[* x’ *]* is reachable from *[* x *]* in the general semantics.

##### **Property2**.

Let *x*,*x*^′^ be states where no process is active. If *[* x’*]* is reachable from *[* x *]* in the general semantics, then *x*^′^ is *not necessarily* reachable from *x* in the stories semantics.

The detailed sketches of proof are in Additional file 2. Property 2 is proved with a counter-example; we give here the main arguments for Property 1. By definition, the occurrence of processes in the stories semantics is more constrained than in the general semantics (due to the additional constraint of exclusivity between the occurrences of processes acting on identical stories). Therefore, if a process occurs in the stories semantics, it can occur in the general semantics. Similarly, if a process stops occurring in the stories semantics, it can stop occurring in the general one as the constraints are equivalent (all the products are present). The application of a process differs in the stories semantics: when the local state of a story changes, it corresponds to a simultaneous production and consumption of the product and reactant EPNs; whereas in the general semantics, the products have to become present first, prior to the (optional) consumption of the reactants. However, as at most one process acting on a story can occur at the same time, this difference in the order of production/consumption cannot introduce spurious transitions in the dynamics: the process has to be fully applied before the implied EPNs can be used for triggering the occurrence of other processes.

Property 2 allows to conclude that cyclic attractors in the stories semantics are not necessarily attractors in the general semantics, although, by Property 1, there is a corresponding transient cycle. Moreover, one can derive that if a state [ *x* ] is a fixed point in the general semantics, then *x* is a fixed point in the stories semantics; the converse is not true when a story embeds a source EPN (see Additional file 2 for a counter example).

**Fig. 7:**
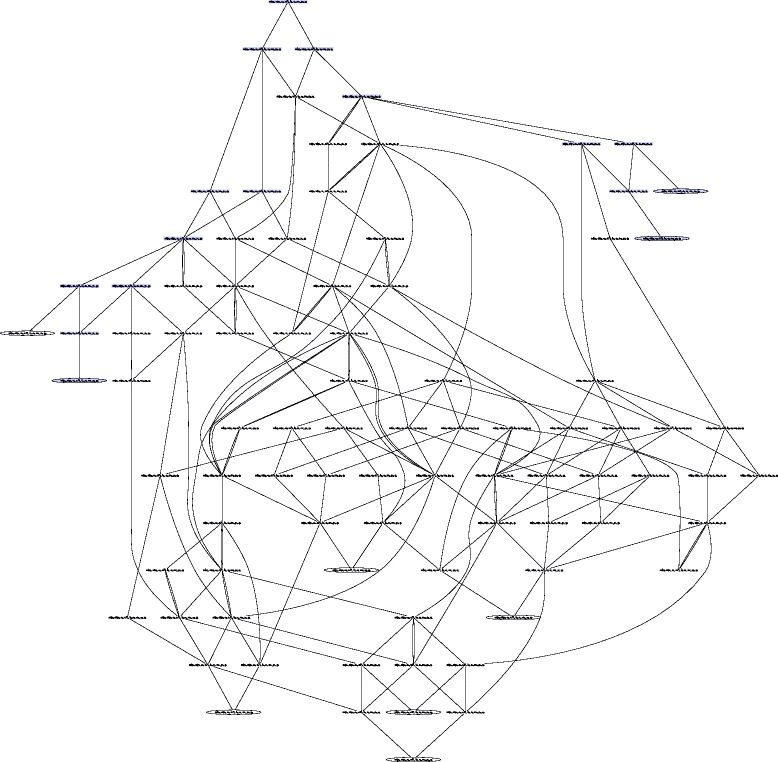
Transition graph for a dynamical model built under the general semantics. Transition graph of the asynchronous automata network of Fig. 3, modeling the SBGN-PD map of Fig. 3. Each node represents a global state of the asynchronous automata network. There is a directed edge from a state *S* to a state *S* ^′^ *iff* *S* ^′^ is reachable from *S*. Circled states are point attractors. States colored in blue are all states present in the transition graph of the asynchronous automata network modeling the same map under the stories semantics (Fig. 8)

**Fig. 8:**
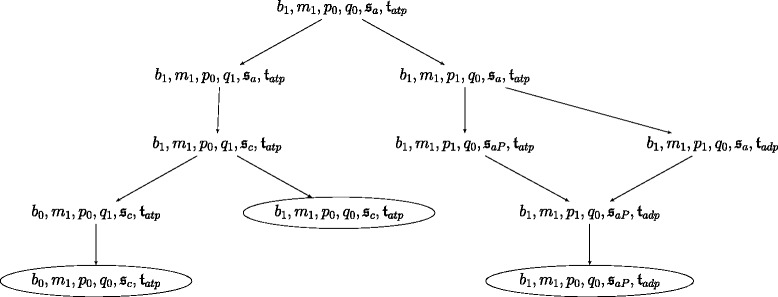
Transition graph for a dynamical model built under the stories semantics. Transition graph of the asynchronous automata network of Fig. 6, modeling the SBGN-PD map of Fig. 6. Each node represents a global state of the asynchronous automata network. There is a directed edge from a state *S* to a state *S* ^′^ *iff* *S* ^′^ is reachable from *S*. Circled states are point attractors

Figures 7 and 8 show the state transition graphs of the ANs modeling our SBGN-PD map example under the general semantics (see Fig. 3) and the stories semantics (see Fig. 6), respectively. States that are point attractors are circled.

**Fig. 9:**
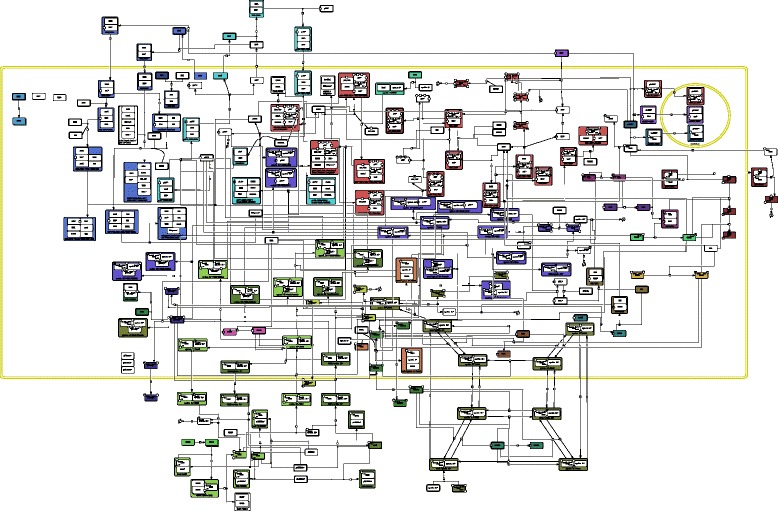
*RB/E2F* map. This map represents the regulation of the cell cycle by E2F/RB. The cell cycle is a succession of four phases (G1, S, G2 and M phases) that are tightly regulated by so-called pocket proteins, whose main representative is the RB protein. The RB protein major function is to inhibit transcription factors belonging to the E2F family, and in particular the E2F1 protein. Diverse cyclin dependent kinases (CDKs) play a key role in the regulation of the cell cycle. In particular, CDKs’ function is to phosphorylate the RB protein, decreasing its inhibiting effect on E2F transcription factors. This map is represented using the SBGN-PD language. EPNs with bold borders constitute the initial state of the map. Every colored EPN belongs to a story, and each color is assigned to a different story

The state transition graph of the model built under the general semantics is composed of 88 states. It has nine attractors, that are all fixed points. Five of these attractors include both local states *aP*_1_ and *c*_1_, meaning that in those states, EPN *aP* and EPN *c* are both present at the same time. Hence in the model built under the general semantics, it is possible to produce both *aP* and *c*, one after the other. There are two possibilities to produce both of them: either process *p* occurs first and is followed by process *q*, or *q* occurs first and is followed by *p*. Either way, the process that occurs first only consumes *a* partially, leaving *a* present so that the second process can occur. Two of the other attractors contain local states *aP*_1_ and *c*_0_, and the last two ones *aP*_0_ and *c*_1_. The former are reached when process *p* occurs first and the latter when process *q* occurs first. In all four cases the first process to occur consumes completely *a*, leaving *a* absent (in the state *a*_0_) and preventing the other process from occurring.

The model built under the stories semantics has only 11 states, three of which are point attractors. This illustrates how the stories semantics induces a lower dimensional dynamics. Two of the attractors contain the local state $\mathfrak {s}_{c}$, meaning that molecular entity *a* is in the state where it is bound to *b*, and only one contains the local state $\mathfrak {s}_{aP}$, meaning that *a* is in a phosphorylated state. As for the point attractors, no other global state contains both local states $\mathfrak {s}_{c}$ and $\mathfrak {s}_{aP}$: EPNs *c* and *aP* belong to the same story, hence they are mutually exclusive (i.e. they cannot be both present at the same time). Among the two point attractors containing the local state $\mathfrak {s}_{c}$, one contains *b*_0_, and the other *b*_1_: when process *q* occurs, it can consume *b* completely or only partially, leaving *b* absent or present, respectively, as *b* does not belong to any story.

The part of the state transition graph of the general semantics that correspond to the dynamics of the stories semantics is highlighted in blue in Fig. 7. It corresponds to the sequences of transitions that lead to total consumption of EPNs belonging to the delimited stories. Because the ordering of production/consumption is different in the stories semantics and requires more steps in the general semantics, there are more blue states than states in the state transition graph of the stories semantics.

### Application to the *RB/E2F* map

In this section, we illustrate how both semantics can be applied to a large network containing 222 nodes, namely the *RB/E2F* map, and how the resulting models can be checked against interesting dynamical properties.

The *RB/E2F* map, represented in Fig. 9, was first published in [5] and made available by the authors at [38] under the CellDesigner format in two versions: the whole map and the map without the transcriptional activations and inhibitions (i.e. the map restricted to proteins). We chose to consider the map restricted to proteins for two reasons: first, CellDesigner’s transcriptional modulations are not SBGN-PD compliant. Second, the protein and the gene parts of the complete maps are distinct from each other, and only the protein part has some effect on the gene part (the proteins activate/inhibit genes, but there are no feedbacks of the genes towards proteins). The map reproduced in Fig. 9 is the map restricted to proteins initially built in CellDesigner. It describes the regulation of the cell cycle focusing on the G1 transition monitored by the retinoblastoma protein (RB) and the E2F transcription factors. The cell cycle is a succession of four phases (G1, S, G2 and M) that are tightly regulated by checkpoints. RB plays a crucial role in ensuring a proper entry into S phase (DNA replication). Its major function is to inhibit E2F1. Diverse cyclin dependent kinases (CDKs) intervene at different moments in the cell cycle and thus play a key role in its regulation. In particular, CDKs phosphorylate RB, slowly releasing its hold on E2F transcription factors. CDKs are only active when associated to their cyclin. There are six major CDKs: CDC2 (also named CDK1), CDK2, CDK3, CDK4, CDK6 and CDK7. CDC2 is associated to cyclin B1, CDK2 to cyclin E1 and cyclin A2, CDK3 to cyclin C, CDK4 and CDK6 to cyclin D1 and CDK7 to cyclin H. As for the E2F transcription factors, they can be divided into two groups: activators (E2F1, E2F2, E2F3a) and inhibitors (E2F3b, E2F4, E2F5, E2F6 and most likely E2F7 and E2F8).

The stimulation by growth factors switches the cells from a quiescent condition (G0) to entry in the cell cycle. Cyclin D1-CDK4,6 complexes are activated and start phosphorylating RB which maintains the G1 checkpoint. As RB starts to be phosphorylated, it frees E2F1 from the inhibitory complex. E2F1 begins to mediate the synthesis of major players of the cell cycle. Cyclin E1-CDK2 complex brings the cells from G1 to the S phase, where DNA is replicated. Following DNA replication and mainly under the action of cyclin A2-CDK2, cells enter a second gap phase, the G2 phase, and finally go through mitosis in the M phase where cyclin B1-CDC2 seems to be one of the main regulators.

#### Models under the general and the stories semantics

We built two models of the *RB/E2F* map, the one under the general semantics and the other under the stories semantics.

The model under the general semantics was built automatically and contained 370 automata.

To build a model under the stories semantics, we first chose a valid set of stories computed from the SBGN-ML file as follows. Since E2Fs and CDKs play a key functional role in the regulation of the cell cycle, we defined one story for each CDK (resp. E2F), each story containing itself all EPNs representing a physical state of the CDK (resp. E2F). We also defined a story beforehand for the p53 protein. Finally, we chose to compute only epn-maximal sets of stories in order to reduce the size of the model as much as possible.

There were only eight epn-maximal sets of stories including all stories defined beforehand, due to three pairs of alternative stories resulting from three different association processes with two reactants (namely, the pairs of reactants {MGA,MAX }, {ATM,NBS1 } and {APC,CDC20 }). All eight sets contained 28 stories for a total of 153 EPNs, out of the 222 EPNs of the map. We chose the valid set focusing on the molecules MGA, ATM and APC, and that is represented in Fig. 9, and built a model under the stories semantics accordingly. This model contained 243 automata.

The analysis of the dynamics of such large models requires advanced techniques to avoid the state space explosion (see the ‘Discussion’ section for more details). Hereafter, we use Mole for this purpose. We show in the next sections how both models can be used to answer biological questions on the network.

#### Building an initial state

In order to check dynamical properties for both models, we first built an initial state that represents a quiescent cell (in G0 phase) just after it has been stimulated by a growth factor (i.e. with CDK4 and CDK6 present). We included in the initial state all EPNs that are inputs of the map. We also included two EPNs that can be produced but belong to cycles: the E2F4 protein in the cytosol and the pRB-E2F1-DP1 complex in the nucleus. The EPNs included in the initial state are shown with a thick black border on the map of Fig. 9.

#### Study of the succession of phases

To illustrate how models built under either semantics can be used to check some interesting dynamical properties on the underlying biological model, we studied the succession of the different phases of the cell cycle in both models. For this sake, we used the software Mole [39]. Mole is a concurrent model analyzer that allows to check for reachability properties in large models where multiple transitions can occur independently, such as those we considered in this work.

##### Phases markers

We associated to each phase of the cell cycle a set of EPNs that are *markers* for this phase. We assume that the system is in a given phase of the cycle at a given time if any of the markers associated to that phase is present at that time. For example, we associated phase G2 to the set of EPNs that represent a complex cyclin B1-CDC2 of the cytosol, with CDC2 phosphorylated or not. G1 and S phases are separated into two periods, early and late, to better characterize transitions.

We define a phase as *reachable* if there exists a state reachable from the initial state such that at least one marker of the phase is present in that state. In a model, a phase marker can be *disabled* by removing all transitions ingoing or outgoing the local state corresponding to the present state of the marker. As for a phase, it can be disabled by disabling all its markers. Hence a phase that has been disabled is no longer reachable.

##### Phases succession in prior-knowledge models

In order to check whether the different phases are reached successively in both models, we first checked if each phase was reachable from the initial state using the Mole tool. As all phases were reachable from the initial state for both models, we checked whether each phase was still reachable when E2F1 was blocked to its initial state. As E2F1 has a central role in the regulation of the cell cycle, preventing any changes in the state of E2F1 should also prevent some phases (if not all) from being reachable in both models. It appeared that all phases but late G1 were still reachable under these conditions in both models.

To test further the validity of our models, we investigated the succession of the different phases in both models. We expected that, apart from early G1, all phases should necessarily be reached successively. Hence we checked, for each phase, whether it was still reachable when its previous phase was disabled. All phases but late G1 and M were still reachable in both models. The fact that early G1 was still reachable under those conditions was expected: indeed, dividing cells can go through multiple cycles without going through G0. However, the models could not reproduce the expected behavior for some of the other phases.

This result shows that the succession of phases observed during the cell cycle cannot be reproduced by the only molecular processes of the map. Indeed, in the obtained dynamical model, the different phases can be reached independently from each other. The sequentiality of phases might be possibly achieved, for instance, by considering the kinetics of processes, or by taking into account additional processes that would enforce synchronization between the pathways of the different phases.

In the scope of this article, we propose to take into account transcriptional effects and investigate the obtained qualitative dynamics by checking if it does reproduce the expected succession of cell cycle phases.

##### Phase succession in models with transcriptional effects

In order to model adequately the succession of phases, we enriched both models by adding known effects of E2F1 on the transcription of some genes whose proteins play a major role in the regulation of the cell cycle. For example, E2F1 is known to upregulate the transcription of CDC2 [5]. As the particular form under which E2F1 is able to regulate CDC2’s expression is not known, we first considered that E2F1 could upregulate CDC2 when associated only to DP1 or when associated to a phosphorylated form of RB, as we know that unphosphorylated RB is an inhibitor of E2F1. We modeled this effect in both models by adding a transition from a state where the molecular entity CDC2 is absent to a state where the CDC2 EPN is present and such that it could be triggered only when E2F1 is in one of the states mentioned above. We added this type of influences on four main regulators of the cell cycle (cyclin E1, cyclin A2, CDC2 and cyclin B1) [5, 40]. Note that these transcriptional effects are not present as such in the CellDesigner version of the map that contains the transcriptional modulations, as these modulations only state that some molecular entities (e.g. E2F1) stimulate or inhibit the transcription of some genes. Hence the physical state under which these molecular entities perform their effect is not specified in this map, and there is no explicit processes linking genes to their corresponding RNA or proteins.

All phases were still reachable from the initial state in the models augmented with transcriptional effects. Yet, no phases but early G1, late S and G2 were reachable when disabling, for each phase, its previous phase. The reachability of late S could be prevented when narrowing the forms of E2F1 able to upregulate the cyclin A2 gene to the complexes where E2F1 is associated only to DP1 or associated to DP1 and RB phosphorylated three times. This suggests that the increase of cyclin A2 after phase G1, that leads to the replacement of cyclin E1 by cyclin A2 in complexes formed of CDK2, might be triggered by the phosphorylation of RB on a third site by the complex cyclin E1-CDK2. As for the succession between late S and G2, it could have been restored in the model by adding a positive influence of cyclin A2-CDK2 on the activation of cyclin B1-CDC2. Such an effect has strong evidence (see [41] for more details), but the precise mechanism remains, to our knowledge, unknown. Hence, adding some transcriptional effects of E2F1 allowed to restore a correct succession for the majority of phases.

Finally, we checked in both augmented models whether two distinct phases of the cell cycle could be reached simultaneously. For each pair of phases, we checked whether there existed a reachable state containing at the same time one marker of the first phase and one marker of the second phase. In the model built under the general semantics, all pairs of phases could be reached simultaneously whereas the couples (early S, late S) and (G2, M) could not be reached simultaneously in the model built under the stories semantics.

The difference observed between the two models is due to the property of mutual exclusiveness of the EPNs of a story. If two markers associated to two different phases belong to the same story, the two phases might not be simultaneously reachable. This last analysis illustrates how the stories semantics can help reasoning about biological processes where successive functional states of some key molecular entities can be linked to biological events that situate at a macro-scale.

## Discussion

### Related work

Notions bearing some similarities with stories can be found in the literature. In [42], authors present a semi-automatic algorithm in order to find *components* in a given pathway. For them, a molecular component corresponds to a biological entity that can appear in the form of different molecular species in the pathway. Hence a component is a species name associated to a set of molecular species that share that name. Their algorithm for inferring pathway components relies on the law of mass conservation, and proceeds iteratively as follows:

- pick arbitrarily a reaction of the pathway not examined yet;
- associate each reactant of the reaction to a different product or to a product split in two parts (by adding new symbols), and memorize these new associations and splits;
- update the associations in the other reactions according to the new associations found and the new splits.

In case of ambiguity when associating the reactants and the products, their algorithm asks the user for the right association.

Stories respecting constraints (i-iv) and molecular components both aim at modeling the changes of states of a particular molecular entity. The main difference between stories and components is that elements of a component are not required to be mutually exclusive. Hence they are not built upon dynamical constraints as for stories, and cannot directly be used within a qualitative semantics in the general case. Let us illustrate this difference on a small example. We consider a pathway containing two processes: the first process is a reaction that transforms *A* into *B*, and the second process is an association between *A* and *B* to form a complex *C*. There would be only one possible story respecting constraints (i-iv): {*A*,*C*}. On the same pathway, the algorithm presented in [42] would automatically find a unique component associated to the set {*A*,*B*,*C*_*A*_,*C*_*B*_} where *C*_*A*_ and *C*_*B*_ are the parts originating from the split of *C*. This component would not be relevant within a dynamics semantics: associated to a unique automaton whose local states would be the elements of the component, *A* and *B* would never be both present at the same time. Hence, the association process would never occur. Therefore the notion of component is not adequate from a dynamics qualitative semantics point of view.

In [5], the authors decompose the *RB/E2F* map into 16 network modules using the Cytoscape plugin BiNoM [43] as follows. First, modules are built by decomposing the *RB/E2F* map network into subnetworks, each focusing on a particular molecular entity. The resulting subnetworks that have more than 30 % overlap are then merged automatically. Finally, the newly-built modules are modified manually to give a biological meaning to each of the networks, which, in most of the cases, corresponds to the different forms that protein can take (phosphorylated, acetylated, in complex, etc.) along with their modifiers (kinases, phosphatases, etc.). The influences between the modules are derived by integrating the influences between the individual molecules within the modules. The resulting network is a modular map of the initial comprehensive map, analogous to an influence graph. Thus, the BiNoM approach focuses on the structure of the complex and detailed network, the SBGN-PD map, by abstracting and simplifying it into an influence network in order to identify possible motifs, such as negative or positive feedback loops, that may be responsible for certain dynamics, but without providing the dynamics. In our case, the stories semantics conserve the level of details of the SBGN-PD model while adding constraints of its dynamical semantics.

### Two semantics to model different types of networks

The general semantics extends BIOCHAM’s semantics by taking into account inhibitions. This semantics can be applied to all biological networks for which precise molecular processes, such as reactions or translocations, are known. That is usually the case for metabolic processes, and for some signaling pathways, such as those presented in the ‘Results’ section.

As for the stories semantics, it can be applied only on networks where physical states of molecular entities can be defined and gathered into stories, that are mainly signaling networks. Using the stories semantics to model metabolic networks would certainly make less sense in general since these networks hardly contain molecules that can be in different states (other than absent/present). Yet modeling some particular metabolic networks under the stories semantics could be imagined. For example, part of the *photosynthetic process* in plants is based on consecutive electron transfers between molecules. One could then build a story focusing on electrons, by regrouping all molecules of unique chains of transfers.

Hence, the general semantics has a broader application range than the stories semantics, as it can be easily applied to metabolic networks. However, as shown for the *RB/E2F* map, the stories semantics allows to build more compact models that are still able to reproduce expected behavior. Moreover, by pruning large portions of the state space entailed by the general semantics, the stories semantics may lead to more realistic models for biological processes that include successive discrete events, such as the phases of the cell cycle.

### Relation between the stories semantics and the Boolean semantics applied to SBGN-AF maps

The stories semantics suits well to signaling networks, where products of reactions are modulators of other reactions, transducing and amplifying an initial signal in this way.

Most molecules of such networks are proteins that can be defined by two states, active and inactive, corresponding in most cases to a normal and a post-translationally modified state (e.g. a phosphorylated state), respectively. These kinds of networks are often represented by influence graphs, where nodes are activities of molecules and arcs are influences between these activities. SBGN-AF is one standard to represent such influence graphs, and a semi-automatic method has been proposed to translate any SBGN-PD map into an SBGN-AF map [44]. Given an SBGN-PD map representing a signaling network, each molecular entity of this network might appear in the form of two different EPNs (representing two different physical states of the same molecular entity) and can be modeled by a story of these two EPNs. Hence the number of automata of the model would be approximately half the number of EPNs of the map. We can then presume that modeling the signaling cascades of a SBGN-PD map representing a signaling network under the stories semantics is analogous to modeling its corresponding (translated) SBGN-AF influence network under a classical Boolean semantics. However, if for simple signaling cascades, modeling the network under the stories semantics might be equivalent to modeling in a more classical way the corresponding influence network, it is not the case for more complicated signaling networks or other types of networks. The *AT*_1*A*_*R-mediated ERK activation* map might well illustrate this difference between modeling simple cascades and more complicated pathways under the stories semantics. The protein G pathway is a simple cascade with reversible processes where one reactant is transformed into one product, and the product of each forward process stimulates the next downstream process. Translating this pathway into SBGN-AF would result in a linear pathway of activities, each of which having a positive influence on the next downstream activity. Modeling this pathway under the stories semantics with three stories of two EPNs as done in the ‘Results’ section would be equivalent to modeling the corresponding SBGN-AF pathway under a Boolean semantics. By contrast, the *β*-arrestin pathway is not a simple cascade, and its translation into SBGN-AF results in a more complicated map than a simple linear pathway. Hence a model of the resultant SBGN-AF pathway built under a Boolean semantics will not be equivalent to a model of the SBGN-PD map built under the stories semantics. The relationship between the stories semantics applied to SBGN-PD maps and the Boolean semantics applied to the corresponding SBGN-AF maps for signaling networks should be deepened in a future work.

### Model-checking, state transition graph, and dynamical properties

Our approach builds dynamical models, i.e., models of state transitions, from SBGN-PD maps. On the resulting models, one can straightforwardly apply generic algorithms for the analysis and inference of dynamical properties, as on any other dynamical model. Most of dynamical analyses have a theoretical computational cost that makes their application to large networks challenging, even though more and more techniques allow to increase their tractability. In the remaining of this section, we give an overview of the use of model-checking and dynamical analyses in systems biology, with mentions to recent computational methods to tackle large models.

Model-checking refers to a wide range of computer science techniques to verify the absence or presence of behaviors within dynamical models. The dynamical properties are typically specified using temporal logic [45], which allow a high-level description of either a trace (succession of transitions), or an execution tree (choices between transitions). Then, generic algorithms have been designed to verify the accordance of a dynamical model with a dynamical property, expressed in temporal logic [46]. Model-checking has been extensively applied to the analysis of biological systems, for instance for gene regulatory networks [47], signalling pathways [48], and models of the circadian clock and the cell cycle [49, 50]. Examples of dynamical properties relevant for biological systems include the reachability of a state where a given molecule is active (e.g., a transcription factor), the reachability of a given differentiated state after a perturbation, the existence of sustained oscillations and their period. All these properties can be analyzed from our models.

Dynamical analyses also allow to make predictions. For instance, the inference of intervention strategies (e.g., the combination of mutations) in order to control the behavior of the system. Recent works have designed algorithms for predicting mutations to prevent or enforce the reachability of particular cell states [30, 51], and methods relying on model-checking for deciphering the reprogramming capability of T-helper cells by determining the inputs of signalling pathways that trigger a change of the cell type [52]. These prediction methods can also be applied to our models.

The computational complexity of model-checking limits, in theory, its applicability to large networks: verifying classical temporal logical formulas, including reachability properties, is PSPACE-complete [53], meaning in practice it is exponential with the number of interacting molecules. A few algorithms for model-checking rely on computing the *state transition graph*, i.e. all the state transitions specified by the dynamical model. For large systems, such a graph may be too large to fit in memory. Therefore, techniques relying on symbolic representations [54] or partial order reductions [55] of such a graph allow to support larger models. Efficient model-checkers (such as NuSMV [56], ITS [57], Mole [39], or PRISM [58]) shipping those techniques are available and can be applied to a large variety of dynamical models, including those introduced in this paper.

Numerous recent works improve the tractability of algorithms for the analysis of biological systems, for instance by exploiting the concurrency (parallelism) of transitions [35], or using abstract interpretation [59], as well as methods to reduce the model size while preserving properties of interest (e.g., [60]). Aforementioned applications of model-checking to systems biology tackle networks ranging from dozens to thousands of interacting molecules.

### Size of the stories and size of the state space

The computation of a valid set of stories for the *RB/E2F* map suggests that there exists a tradeoff between the number of stories of a valid set and the size of the stories of the set. Indeed, we computed a valid set of stories maximizing the total number of distinct EPNs involved in a story for the *RB/E2F* map. This set contained 42 stories for a total of 167 EPNs, to be compared to the 28 stories for 153 EPNs of the valid set computed by defining stories beforehand (see the ‘Results’ section). Hence, in this example, increasing the total number of EPNs involved in a story led to smaller stories. This tradeoff can be illustrated on a simple example. Let us consider a map with two processes that model the reactions *A*→*B* and *B*+*C*→*D*. Given constraints (i-v), the map has two maximally (in the sense of inclusion) valid sets of stories: {{*A*,*B*},{*C*,*D*}} and {{*A*,*B*,*D*}}. Only the first set maximizes the total number of EPNs (i.e. contains four EPNs). However, its ratio EPN/stories is smaller than for the second set: it contains two stories, compared to the second set that contains only one story of three EPNs.

Building models under the stories semantics induces a reduction of the number of automata and subsequently a large reduction of the size of the state space. Hence dynamics that may be intractable for an exhaustive analysis under the general semantics may become tractable under the stories semantics.

Considering the stories semantics as an abstraction of the quantitative population semantics [14], its property of mutual exclusiveness of the EPNs of a story comes down to force synchronous transitions between all molecules of a population (or to have populations made of only one molecule). Hence the stories semantics prunes all the traces of the (abstracted) population dynamics where a molecule can be simultaneously into two states.

### Future work

#### Adding constraints

Some additional constraints could be considered in order to define stories. For example, we do not consider the case where a story contains two EPNs one being a reactant of a process and the other being the only stimulator of the same process. Modeling a map with such a story under the stories semantics would prevent the process from occurring. Hence constraints forbidding such cases will be added in a future work.

#### Formalizing models into SBML-qual

The Systems Biology Markup Language (SBML) [61] is a standard to store and exchange systems biology models built upon reaction networks. SBML-qual [62] is an additional package that allows to store qualitative models such as Boolean Networks or Petri Nets. Models built under the general semantics can be stored in the SBML-qual format and a tool to convert asynchronous AN models built under the general semantics into this format is under development. However, SBML-qual does not yet allow to encode models containing variables that take their value onto unordered domains. Hence automata representing stories cannot be properly encoded within the current version of SBML-qual.

#### Software development

**Fig. 10:**
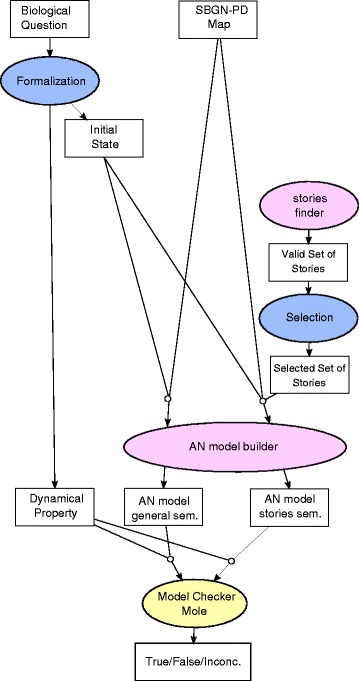
Workflow of the method. *Rectangles* represent objects and ellipses tasks. *Pink* processes are those developed as part of this work; *blue* processes are users’ interventions; *yellow* processes are tools publicly available that were used for this study

A user-friendly software taking into account the whole framework presented in this article (see Fig. 10) is under development.

This software should allow to compute all valid sets of stories respecting constraints on the content of stories and on maximality defined by the user thanks to a GUI, and build AN dynamical models automatically.

## Conclusions

In this article we propose two qualitative dynamics semantics for SBGN Process Description maps, that represent a particular class of reaction networks. Besides extending existing generic interpretations of reaction networks with Boolean logic, we introduce the new concept of stories, that allows to focus on physical states of molecular entities rather than on the entities themselves. The dynamics in stories semantics have a lower dimension than the general one and prune multiple behaviors (which can be considered as spurious) by enforcing the mutual exclusivity between the activities of the different EPNs of a given story. Moreover, the stories semantics leads to more realistic models when discrete successive events can be underlined in the biological process to be modeled. We illustrate these two semantics applying them to a large network. By performing a dynamical analysis of the RB/E2F pathway, that contains more than 200 nodes, we show how the qualitative approach allowed us to propose improvement of the initial model.

## Methods

### From SBGN-PD to automata networks

We detail here the formal encoding of SBGN-PD to Automata Networks, with the two semantics (general and stories) that we have introduced in this paper. Note that both semantics can be expressed within the same encoding: the encoding of the general semantics is a special case of encoding of the stories semantics where the chosen set of stories is empty.

We use the following notations for referring to a given SBGN-PD model:

- $\mathcal {E} = \{e_{1}, \cdots, e_{n}\}$ is the finite set of EPNs;
- $\mathcal {P} = \{p_{1}, \cdots, p_{m}\}$ is the finite set of processes;
- For each process $p\in \mathcal {P}$, in(*p*) (resp. out(*p*)) denotes the set of EPNs – except sinks and sources EPNs – that are reactants (resp. products) of *p*.

#### Logic of modulations

As described in the ‘Background’ section, the modulation of SBGN-PD processes is specified using modulation arcs that link either an EPN or a logical operator to the modulated process. Modulations can be split in three classes: *necessary stimulations*, denoted by req(*p*) – describing conditions that are required for the process to occur; *catalyses* and *stimulations*, denoted by act(*p*) – describing conditions that activate the process; and *inhibitions*, denoted by inh(*p*) – describing conditions that inhibit the process. When the effect of a modulation is unknown, SBGN-PD allows to specify it with a generic *modulation*.

To each node *n* at the origin of a modulation arc, we associate a Boolean formula logic(*n*) for the satisfaction of *n*. Boolean formulae are constructed with classical AND (∧) and OR (∨) logical operators upon literals denoting the presence of an EPN. Hereafter, in(*n*) denotes the set of parent nodes of the node *n*:

$${\textsf{logic}}(n) \stackrel{\Delta}=\left\{ \begin{array}{ll} e & \text{if}~ n = e \in \mathcal{E}\\ \bigwedge_{m\in {\textsf{in}}(n)} {\textsf{logic}}(m) & \text{if}{n}\text{is an AND node}\\ \bigvee_{m\in {\textsf{in}}(n)} {\textsf{logic}}(m) & \text{if}{n}\text{is an OR node} \end{array} \right. $$

Finally, mod(*p*) defines the Boolean formula that must be satisfied in order to make the process *p* to occur. In the case where process *p* has multiple modulating arcs, several different interpretations can be derived. In the scope of this paper, we use a permissive interpretation that (i) requires the satisfaction of all the necessary stimulations; (ii) if any, requires at least one stimulation satisfied, or at least one inhibition *not* satisfied:

If *act*(*p*)=inh(*p*)=*∅*, ${\textsf {mod}}(p)\stackrel {\Delta }= \textstyle \bigwedge _{n{\in }\textsf {req}(p)}{\textsf {logic}}(n)$; otherwise,

$$\begin{array}{*{20}l} {\textsf{mod}}(p) & \stackrel{\Delta}= \textstyle\bigwedge_{n{\in}\textsf{req}(p)} {\textsf{logic}}(n)\\ \wedge &\left(\textstyle\bigvee_{n{\in}\textsf{act}(p)} {\textsf{logic}}(n) \vee \bigvee_{n{\in}\textsf{inh}(p)} {\neg}\textsf{logic}(n) \right). \end{array} $$

By convention, $\bigwedge _{\emptyset } = \mathbf {true}$ and $\bigvee _{\emptyset } = \mathbf {false}$.

##### **Example**.

In Fig. 4, the logic of the modulation of process *p* is mod(*p*)=logic(*m*)=*m*.

#### Stories declaration

A *story*${\mathfrak {S}}$ is a subset of the set of EPNs $\mathcal {E}$ excluding sinks and sources EPNs satisfying the following conditions (cf. constraints (i)-(iv) of the ‘Results’ section):

- $\forall e,f\in {\mathfrak {S}}, e\neq f, \exists p^{1},\dots,p^{k}\in \mathcal {P}$ such that:
  - $\forall i\in \{1,\dots,k-1\}, \exists g \in {\mathfrak {S}}: g \in ({\mathsf {out}}(p^{i})\cup {\mathsf {in}}(p^{i})) \cap ({\mathsf {out}}(p^{i+1}) \cup {\mathsf {in}}(p^{i+1}))$
  - *e*∈*in*(*p*^1^)∪*out*(*p*^1^) and *f*∈*in*(*p*^*k*^)∪*out*(*p*^*k*^).
- $\forall p\in \mathcal {P}, {\mathsf {out}}(p)\cap {\mathfrak {S}}\neq \emptyset \Rightarrow ({\mathsf {in}}(p)=\emptyset \vee {\mathsf {in}}(p)\cap {\mathfrak {S}}\neq \emptyset)$,
- $\forall p\in \mathcal {P}, |{{\mathsf {in}}(p)\cap {\mathfrak {S}}} \leq 1|$,
- $\forall p\in \mathcal {P}, |{{\mathsf {out}}(p)\cap {\mathfrak {S}}} \leq 1|$.

A set of stories ${\mathbb {S}} =\{{\mathfrak {S}}_{A}, \dots, {\mathfrak {S}}_{Z} \}$ is *validiff* the stories are pairwise disjoint: $\forall {\mathfrak {S}}_{A},{\mathfrak {S}}_{B}\in {\mathbb {S}}, {\mathfrak {S}}_{A} \cap {\mathfrak {S}}_{B} = \emptyset $. We note the union of all the stories ${\cup \mathbb {S}}\stackrel {\Delta }= \bigcup _{{\mathfrak {S}}\in {\mathbb {S}}}{\mathfrak {S}}$, and the set of stories that are involved in a process *p* with ${\mathbb {S}}(p) = \{{\mathfrak {S}}\in {\mathbb {S}}\mid {\textsf {in}}(p)\cap {\mathfrak {S}}\neq \emptyset $$\vee {\textsf {out}}(p)\cap {\mathfrak {S}}\neq \emptyset \}$.

We define a symmetric irreflexive relation $\# \subset \mathcal {P}\times \mathcal {P}$ ($\forall p,q\in \mathcal {P}, p\# q \Rightarrow q\# p \wedge p\neq q$) such that: for each pair of two different processes $p,q\in \mathcal {P}$, if ${\mathbb {S}}(p)\cap {\mathbb {S}}(q)\neq \emptyset $, *p#q*. This relation can be read as *conflicts*: *p#q* means that *p* and *q* should not occur simultaneously.

##### **Example**.

In Fig. 6, ${{S}} = \{\mathfrak {s}, \mathfrak {t}\}$ with $\mathfrak {s}=\{a,aP,c\}$ and $\mathfrak {t}=\{adp,atp\}$. ${\cup \mathbb {S}} = \{a,aP,c,adp,atp\}$; ${\mathbb {S}}(p) = \{\mathfrak {s}, \mathfrak {t}\}$ and ${\mathbb {S}}(q) = \{\mathfrak {s}\}$. Because ${\mathbb {S}}(p)\cap {\mathbb {S}}(q)=\mathfrak {s}$, *p* and *q* are in conflict, i.e., *p#q*.

#### Encoding of automata

##### (1) For each EPN not belonging to any story

$e\in \mathcal {E}\setminus {\cup \mathbb {S}}$ that is neither a source nor a sink EPN, e∈*Σ* with *S*(e)={e_0_,e_1_}.

##### (2) For each story

${\mathfrak {S}}\in {\mathbb {S}}$, we define an automaton ${\mathfrak {s}}\in \Sigma $, with $S({\mathfrak {s}}) = \{ {{\mathfrak {s}}}_{\textsf {e}} \mid e\in {\mathfrak {S}}\} \cup \{ {\mathfrak {s}}_{\emptyset }\}$, where ${\mathfrak {s}}_{\emptyset }$ represents the inactivity of story ${\mathfrak {S}}$.

##### (3) For each process

$p\in \mathcal {P}$, we define an automaton p∈*Σ*, with *S*(p)={p_0_,p_1_}, except for simple cases where no additional automaton is required for controlling the dynamics. It is the case when *p* has no conflict ($\nexists q\in \mathcal {P}: p\# q$) and either in(*p*)=*∅* (no consumption); or in(*p*)={*e*} and out(*p*)=*∅* (single consumption); or in(*p*)={*e*} and out(*p*)={*f*} with $\{e,f\}\subseteq {\mathfrak {S}}$, where ${\mathfrak {S}}\in {\mathbb {S}}$ (simple change of story state). In those cases, the conditions for the occurrence of process *p* are directly embedded in the transition conditions within automata of the concerned EPNs.

#### Encoding of modulations

The logic of process modulations is translated as follows. Given a process $p\in \mathcal {P}$, having a set of modulations mod(*p*), we write DNF(mod(*p*)) the representation in disjunctive normal form of the Boolean formula mod(*p*). Hence, DNF(mod(*p*)) is a set of clauses, where each clause is a set of literals denoting the presence or absence (noted ¬) of the associated EPN. We define ls(*x*) as the local states that match with the literal *x*, and cond(*p*) the set of sets of local states that satisfy DNF(mod(*p*)). We recall that an EPN belonging to a story is absent if any of the other EPNs of the story is present:

$${\textsf{ls}}(x) \stackrel{\Delta}=\left\{ \begin{array}{ll} \{{\mathsf{e}}_{1}\} & \text{if}~x = e, e \in\mathcal{E}\setminus \cup\mathbb{S}\\ \{{\mathsf{e}}_{0}\} & \text{if}~ x = \neg e, e \in\mathcal{E}\setminus\cup\mathbb{S}\\ \{{{\mathfrak{s}}}_{\textsf{e}}\} & \text{if }x = e, e\in{\mathfrak{S}},{\mathfrak{S}}\in{\mathbb{S}}\\ \{{{\mathfrak{s}}}_{\textsf{f}} \mid f\in{\mathfrak{S}}, f\neq e\} & \text{if}~x = \neg e, e\in{\mathfrak {S}},{\mathfrak{S}}\in{\mathbb{S}} \end{array} \right. $$

$$\begin{array}{*{20}l} \textsf{cond}(p)&\stackrel{\Delta}=\textstyle\bigcup_{cl{\in}\textsf{DNF}({\textsf{mod}}(p))} \prod_{x\in cl} {\textsf{ls}}(x). \end{array} $$

#### Encoding of transitions

Transitions are defined for each $p\in \mathcal {P}$ as follows:

If in(*p*)=*∅* and $\nexists q\in \mathcal {P}:p\# q$ (*p* has no conflict), for each enabling condition *ℓ*∈cond(*p*), for each *f*∈out(*p*), if *f* belong to a story ${\mathfrak {S}}$, then ${\mathfrak {s}}_{\emptyset }\overset {\ell }{\rightarrow }{\mathfrak {s}}_{\text {f}}\in T$, else, $\text {f}_{0}\overset {\ell }{\rightarrow }\text {f}_{1}{\ell }\in T$.

Otherwise, if out(*p*)=*∅*, in(*p*)={*e*}, and $\nexists q: p\# q$, for each *ℓ*∈cond(*p*), if *e* belongs to a story ${\mathfrak {S}}$, then ${\mathfrak {s}}_{\textsf {e}}\overset {\ell }{\rightarrow }\mathfrak {s}_{\emptyset } \in T$, else, $\textsf {e}_{1}\overset {\ell }{\rightarrow }\textsf {e}_{0}\in T$.

Otherwise, if in(*p*)={*e*} and out(*p*)={*f*} with *e* and *f* in the same story ${\mathfrak {S}}$, and $\nexists q: p\# q$, for each *ℓ*∈cond(*p*), $\mathfrak {s}_{\textsf {e}}\overset {\ell }{\rightarrow }\mathfrak {s}_{\textsf {f}} \in T$.

Otherwise, in the general case, with

$$\begin{array}{@{}rcl@{}} \begin{array}{ll} \mathsf{ready}(p) &\stackrel{\Delta}= \left\{ {\textsf{e}}_{1} \mid e\in {\mathsf{in}}(p)\setminus{\cup\mathbb{S}}\right\} \\ & \qquad\cup \left\{{{\mathfrak{s}}}_{\textsf{e}} \mid {\mathsf{in}}(p)\cap{\mathfrak{S}}=\{e\}, {\mathfrak {S}}\in{\mathbb{S}}\right\} \\ & \qquad\cup \left\{ {{\mathfrak{s}}}_{\emptyset} \mid {\mathsf{in}}(p)=\emptyset, {\mathsf{out}}(p)\cap{\mathfrak {S}}\neq\emptyset, {\mathfrak{S}}\in{\mathbb{S}}\right\} \\ & \qquad\cup \left\{ {\textsf{q}}_{0} \mid p\# q\right\}\\ \mathsf{done}(p) &\stackrel{\Delta}= \left\{ {\mathsf{e}}_{1} \mid e \in {\mathsf{out}}(p)\setminus{\cup\mathbb{S}} \right\}\\ & \qquad\cup \left\{ {{\mathfrak{s}}}_{\textsf{f}} \mid {\mathsf{out}}(p)\cap{\mathfrak{S}}=\{f\},{\mathfrak{S}}\in{\mathbb{S}}\right\}\\ & \qquad\cup \left\{ {{\mathfrak s}}_{\emptyset} \mid {\mathsf{in}}(p)\cap{\mathfrak{S}}\neq\emptyset,\right.\\ & \qquad\qquad\qquad \left. {\mathsf{out}}(p)\cap{\mathfrak{S}}=\emptyset,{\mathfrak{S}}\in{\mathbb{S}}\right\}, \end{array} \end{array} $$

where ${\mathfrak {S}}$ is the automaton of story ${\mathfrak {S}}$, process activation for each *ℓ*∈cond(*p*),$\textsf {p}_{0} \xrightarrow {\ell \cup \mathsf {ready}(p)}\textsf {p}_{1} \in T$ production for each *f*∈*out*(*p*) such that $f\notin {\cup \mathbb {S}}$,$\textsf {f}_{0} \xrightarrow {\{ {\mathsf {p}}_{1} \}} \mathsf {f}_{1}\in T$. consumption for each *e*∈*in*(*p*) such that $e\notin {\cup \mathbb {S}}$,${\textsf {e}_{1}} \xrightarrow {\{ \mathsf {p}_{1} \} \cup \textsf {done}(p)} \textsf {e}_{0}\in T$. stories for each ${\mathfrak {S}}\in {\mathbb {S}}$:if there exists $e\in {\mathsf {in}}(p)\cap {\mathfrak {S}}$, if ${\textsf {out}}(p)\cap {\mathfrak {S}}=\left \{f\right \}$, $\mathfrak {s}_{\textsf {e}} \xrightarrow {\mathsf {p}_{1}}\mathfrak {s}_{\textsf {f}} \in T$;otherwise ($\textsf {out}(p)\cap {\mathfrak {S}}=\emptyset $), $\mathfrak {s}_{\textsf {e}} \xrightarrow {\mathsf {p}_{1}} \mathfrak {s}_{\emptyset }\in T$.If in(*p*)=*∅*, and there exists $f\in {\textsf {out}}(p)\cap {\mathfrak {S}}$, then $\mathfrak {s}_{\emptyset }\overset {\mathsf {p}_{1}}{\rightarrow }\mathfrak {s}_{\textsf {f}}\in T$. process de-activation

$\mathsf {p}_{1} \xrightarrow {\textsf {done}(p)} \mathsf {p}_{0}\in T$.

The complexity of the encoding is polynomial in the number of EPNs and processes, and exponential with the number, per process, of inhibitions belonging to a story. The combinatorics is due to the negation of the presence of a story at a particular state, which involves enumerating all other states of the story. Such a complexity can be drastically reduced by allowing Boolean formulae for specifying cond(*p*), instead of lists of local states.

### Identifying stories

Valid sets of stories meeting constraints on the content of stories or on maximality can be identified automatically from an SBGN-PD map (in the SBGN-ML format). We use a declarative programming approach, Answer-Set Programming (ASP) [63] to specify constraints (i-iv) that stories must satisfy and the following optional additional constraints: constraint (v), possible seeds of stories, and epn-maximality. Then, ASP solvers such as [64] allow a fast exploration of the state space to retrieve all valid sets of stories considering the compound graph of the map. Epn-maximality is encoded using the *#maximize* keyword available in clingo, that allows to obtain only answer sets maximizing the number of atoms specified by the *#maximize* statement. Finally, final sets of stories can be retrieved by filtering *a posteriori* the valid sets of stories.

### Analysis of automata networks dynamics

Given an Automata Network (*Σ*,*S*,*T*), and using its asynchronous semantics as defined in previous sub-section, we define the following dynamical features: State reachability Given two states *s*,*s*^′^∈*S*, *s*^′^ is *reachable* from *s*, noted *s*→^∗^*s*^′^*iff* either *s*→*s*^′^ or there exists a state *s*^″^∈*S* such that *s*→*s*^″^ and *s*^′^ is reachable from *s*^″^. By convention, *s*→^∗^*s*. Reachable state space Given a state *s*∈*S* the reachable state space *X*(*s*) from *s* is the set of states that can be reached from *s*: *X*(*s*)={*s*^′^∈*S*∣*s*→^∗^*s*^′^}. Attractors An attractor *A*⊆*S* is a *minimal* set of states such that: ∀*s*∈*A*,*X*(*s*)⊆*A*. If *A* contains only one state, *A*={*s*}, *s* is called a *point attractor* (or *fixed point*); otherwise *A* is a *cyclic attractor*.

Given an SBGN-PD map, an Automata Network (*Σ*,*S*,*T*) modeling that map under either semantics and a global initial state of the Automata Network, we also define the following additional features that are used for the analysis of the *RB/E2F* map (see the ‘Results’ section). Phase and markers A *phase* is a set of EPNs of the map, and these EPNs are called the *markers* of that phase. Presence of a marker A marker *e* is *present* in a state *s*∈*Siffe*_1_∈*s* if *e* does not belong to any story, and $\mathfrak {s}_{e} \in s$ if *e* belongs to story $\mathfrak {S}$. Phase reachability Given a phase *p* and a state *s*∈*S*, *p* is *reachable* from *siff* there exists at least one marker *e*∈*p* and a state *s*^′^∈*S* s.t. *s*→^∗^*s*^′^ and *e* is present in *s*^′^. Phase *p* is *reachable* if it is reachable from the global initial state. Phases simultaneous reachability Given two phases *p* and *q* and a state *s*∈*S*, *p* and *q* are *simultaneously reachable* from *siff* there exist two markers *e*∈*p*, *f*∈*q* and a state *s*^′^∈*S* s.t *s*→^∗^*s*^′^, *e* is present in *s*^′^ and *f* is present in *s*^′^. Phases *p* and *q* are *simultaneously reachable* if they are simultaneously reachable from the global initial state.

We used the software Pint [65] and Mole [39] to compute the various reachability properties. Pint takes as input models of automata networks (ANs). Pint has been used to reduce the model dynamics with respect to reachability properties: it guarantees to preserve the traces for the concerned reachability, but removes unnecessary transitions, which can reduce considerably the dynamics to explore for the model checking. Then, for each reduced model, we checked the reachability property using Mole. The reduction step, relying on the AN framework, was mandatory to make the reachability computations tractable. Mole takes as input models of (1-bounded) Petri nets and computes their *unfolding*, that is a partial order representation of the possible sequences of transitions. The Petri nets models have been generated by Pint using the encoding of [35]. All but one reachability property of the *RB/E2F* map case study are tractable on a computer with 16GB of RAM. The non-tractable reachability property is the simultaneous reachability of the couple (G2, M) for the model built under the stories semantics and augmented with transcriptional effects. However, this reachability property is False in the model built under the stories semantics without transcriptional effects. Therefore, since the dynamics of the model augmented with transcriptional effects is a restriction of the dynamics of the model without these effects, this property is also False for the model augmented with transcriptional effects.

### Conversion from the CellDesigner format to the SBGN-ML format

The CellDesigner file for the *RB/E2F* map was converted to an SBGN-ML file using the export to SBGN-ML function of CellDesigner.

### Workflow

All commands necessary to carry out the various analyses presented in this article are available at https://github.com/pauleve/sbgnpd2an-suppl.

Figure 10 presents the workflow of the method introduced in this paper. From any SBGN-PD map stored in the SBGN-ML format, valid sets of stories can be computed automatically. Then two models can be built: a model under the general semantics directly from the map, and a model under the stories semantics taking as input the map and a valid set of stories chosen by the user. The models can then be checked against dynamical properties using state of the art model checkers, such as Mole.

## Abbreviations

AN, automata network; ASP, answer set programming; CDK, cyclin dependent kinase; EPN, entity pool node; ODE, ordinary differential equation; PN, process node; RB, retinoblastoma protein; SBGN, systems biology graphical notation; SBGN-AF, systems biology graphical notation activity flow language; SBGN-ER, systems biology graphical notation entity relationship language; SBGN-ML, systems biology graphical notation markup language; SBGN-PD, systems biology graphical notation process description language; SBML, systems biology markup language; SBML-qual, systems biology markup language qualitative models; SBO, systems biology ontology

## Additional files

Additional file 1 Encoding of an asynchronous automata network into the Petri net formalism. Provides the translation of the AN of Fig. 2 into the Petri net formalism. Each local state of the AN is encoded into a place in the Petri net, and each transition of the AN is encoded into one transition in the PN, with one input and one output arc. Transition conditions of the AN are encoded under the form of read arcs in the Petri net. (PDF 88.7 kb)

Additional file 2 Relationship between stories and general semantics. Provides detailed sketches of proof for the properties relating the stories and the general semantics. (PDF 251 kb)
