# Supplementary material for: Qualitative dynamics semantics for SBGN process description
Source: BMC Syst Biol. 2016 Jun 16;10:42. doi: 10.1186/s12918-016-0285-0 (PMC4910245; doi:10.1186/s12918-016-0285-0)
Supplement: Additional file 1 — Encoding of an asynchronous automata network into the Petri net formalism. Provides the translation of the AN of Fig. 2 into the Petri net formalism. Each local state of the AN is encoded into a place in the Petri net, and each transition of the AN is encoded into one transition in the PN, with one input and one output arc. Transition conditions of the AN are encoded under the form of read arcs in the Petri net. (PDF 88.7 kb) [file 12918_2016_285_MOESM1_ESM.pdf]

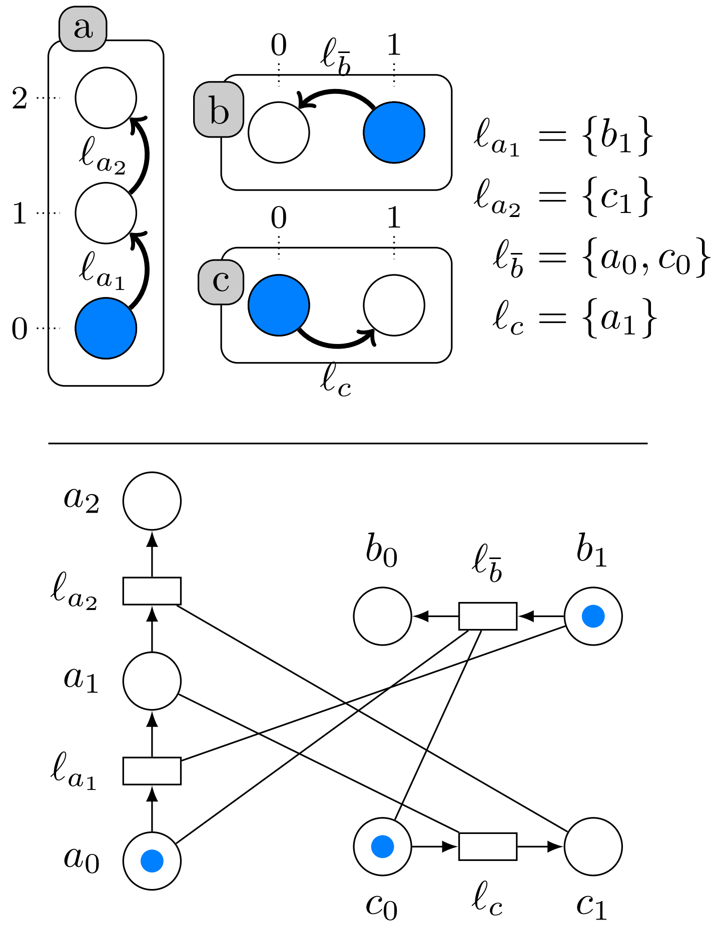

Figure 1: **Encoding of an automata network into a Petri net.** Top: an example of automata network (AN). The global initial state $\langle a_0, b_1, c_0 \rangle$ is represented in blue. Bottom: the corresponding Petri net (PN). Circles represent places, rectangles represent transitions, arrows represent input and output arcs (incoming to and outgoing from transitions, respectively), undirected edges represent read arcs, and blue points represent tokens. A marking of the PN is any distribution of tokens in the places. Each local state of each automaton of the AN is encoded into a place in the PN, and each transition of the AN is encoded into a transition in the PN with one input and one output arc. Conditions of the local transitions of the AN are encoded into read arcs in the PN. Finally, the initial marking of the PN corresponding to the initial global state of the AN is represented in blue. A transition can be fired if and only if all places linked to it by an input arc or a read arc have at least one token. When a transition is fired, all tokens are removed from every input place, and one token is added to every output place. In PN, places are *a priori* independent from each other, therefore the components a, b and c remain implicit. They can be uncovered only by additional computations on the structure and dynamics of the net to prove that the related places are actually mutually exclusive.
