# Supplementary material for: Qualitative dynamics semantics for SBGN process description
Source: BMC Syst Biol. 2016 Jun 16;10:42. doi: 10.1186/s12918-016-0285-0 (PMC4910245; doi:10.1186/s12918-016-0285-0)
Supplement: Additional file 2 — Relationship between stories and general semantics. Provides detailed sketches of proof for the properties relating the stories and the general semantics. (PDF 251 kb) [file 12918_2016_285_MOESM2_ESM.pdf]

Supplementary material
Relationship between stories and general semantics

The purpose of this supplementary material is to give detailed sketches of proof on the relationships between the stories and general semantics.

We consider a global automata network (Σ, S, T) resulting from the encoding of a SBGN-PD map with EPNs $\mathcal{E}$, processes $\mathcal{P}$, and stories $\mathbb{S}$.

We use the definitions and notations of the method section from the main text, at the exception of transition simplifications listed at the beginning of the sub-section *Encoding of transitions*: we consider here only the general case. In order to differentiate the value of functions which depends on the defined stories $\mathbb{S}$, we append the sign * to functions used to model the general semantics (e.g., $\mathsf{ready}^\star(p)$, $\mathsf{done}^\star(p)$, etc.).

Definition 1 ($[\![\cdot]\!]$)**.** *Given a state $x \in S$, $[\![x]\!]$ is the corresponding state in the general semantics where local states of stories are replaced by the matching local states of EPNs. $[\![x]\!]$ verifies the following properties:*

1. $\forall p \in \mathcal{P}, \forall i \in \{0,1\}, \mathsf{p}_i \in x \Leftrightarrow \mathsf{p}_i \in [\![x]\!]$*;*
2. $\forall e \in \mathcal{E} \setminus \cup\mathbb{S}, \forall i \in \{0,1\}, \mathsf{e}_i \in x \Leftrightarrow \mathsf{e}_i \in [\![x]\!]$*;*
3. $\forall \mathfrak{S} \in \mathbb{S}, \forall e \in \mathfrak{S}, \mathfrak{s}_\mathsf{e} \in x \Leftrightarrow \mathsf{e}_1 \in [\![x]\!]$ *and* $\mathfrak{s}_\mathsf{e} \notin x \Leftrightarrow \mathsf{e}_0 \in [\![x]\!]$.

Lemma 1. *Given a state $x \in S$, if a process $p \in \mathcal{P}$ can get activated in the stories semantics, it can get activated in $[\![x]\!]$ in the general semantics.*

Proof. If $p \in \mathcal{P}$ can get activated in x, then there exists $\ell \in \mathsf{cond}(p)$ such that $\ell \subseteq x$ and $\mathsf{ready}(p) \subseteq x$.

We remark that $\mathsf{ready}^\star(p)$ is composed only of local states of the form e_1 with $e \in \mathsf{in}(p) \cap \mathcal{E}$. By definition of ready, if $e \notin \cup\mathbb{S}$, then $\mathsf{e}_1 \in \mathsf{ready}(p)$, therefore $\mathsf{e}_1 \in [\![x]\!]$; otherwise, if $\exists \mathfrak{S} \in \mathbb{S}$ such that $e \in \mathfrak{S}$, by definition, $\mathfrak{s}_\mathsf{e} \in \mathsf{ready}(p)$, and, by $[\![\cdot]\!]$ definition, $\mathfrak{s}_\mathsf{e} \in x \Rightarrow \mathsf{e}_1 \in [\![x]\!]$; therefore $\mathsf{ready}^\star(p) \subseteq [\![x]\!]$.

Let $cl \in \mathsf{DNF}(\mathsf{mod}(p))$ such that $\ell \in \prod_{d \in cl} \mathsf{ls}(d)$. We show that $\exists \ell^\star \in \prod_{d\in cl} \mathsf{ls}^\star(d)$ with $\ell^\star \subseteq [\![x]\!]$. For each $d \in cl$, if $d = e$ or $d = \neg e$ with $e \in \mathcal{E} \setminus \cup\mathbb{S}$, then $\mathsf{ls}(d) = \mathsf{ls}^\star(d)$; otherwise if $d = e$ with $e \in \mathfrak{S}$ where $\mathfrak{S} \in \mathbb{S}$, then $\mathsf{ls}(d) = \{\mathfrak{s}_\mathsf{e}\}$ and $\mathsf{ls}^\star(d) = \{\mathsf{e}_1\}$, thus, by definition of $[\![\cdot]\!]$, as $\mathfrak{s}_\mathsf{e} \in x$, $\mathsf{e}_1 \in [\![x]\!]$; finally, if $d = \neg e$ with $e \in \mathfrak{S}$ where $\mathfrak{S} \in \mathbb{S}$, $\mathsf{ls}(d) = \{\mathfrak{s}_\mathsf{f} \mid f \in \mathfrak{S}, f \neq e\}$ and $\mathsf{ls}^\star(d) = \{\mathsf{e}_0\}$, as $\ell \subseteq x$, $\mathfrak{s}_\mathsf{e} \notin x$, thus by definition of $[\![\cdot]\!]$, $\mathsf{e}_0 \in [\![x]\!]$. Hence, $\ell \subseteq x \Longrightarrow \exists \ell^\star \in \mathsf{cond}^\star(p) : \ell^\star \subseteq [\![x]\!]$. □

Definition 2 (Coherent state)**.** *A state $x \in S$ is coherent if no active process is in conflict with another active process: $x \in S$ is* coherent *if and only if $\forall p \in \mathcal{P}, \mathsf{p}_1 \in x \Rightarrow \forall q \in \mathcal{P} : p\#q, \mathsf{q}_0 \in x$.*

Property 1. *Let $x, x' \in S$ be states where no process is active. If x' is reachable from x in the stories semantics, then $[\![x']\!]$ is reachable from $[\![x]\!]$ in the general semantics.*

Proof sketch. Because x has no process active, it is coherent. By lemma 1, any process activation in the stories semantics can be executed in the general semantics.

Then, we show that the sequence of transitions in the stories semantics from a state y where p has just been activated and that lead to the de-activation of p has a counter-part in the general semantics: it is necessary and sufficient that all the *production* transitions and all the *stories* transitions conditioned with $\{\mathsf{p}_1\}$ are executed. One can remark that (1) all those transitions are independent from each other, therefore, they can be executed in any order; (2) the *production* and *consumption* transitions of the stories semantics are the same in the general semantics. Furthermore, the reachability of a state y' where $\mathsf{done}(p) \subseteq y'$ is inevitable in the stories semantics:

- for each $e \in \mathsf{out}(p) \setminus \cup\mathbb{S}$, e_1 is always eventually reachable as long as p_1 is present. Indeed the automaton e has only two states $0, 1$, and the transition $\mathsf{e}_0 \to \mathsf{e}_1$ is conditioned by p_1 only.
- for any story $\mathfrak{S} \in \mathbb{S}$ with $\mathsf{out}(p) \cap \mathfrak{S} = f$, $\mathfrak{s}_\mathsf{f}$ is always eventually reachable as long as p_1 is present. Indeed, the automaton $\mathfrak{s}$ is either in the same state as in y, or in $\mathfrak{s}_\mathsf{f}$, as no other process acting on $\mathfrak{S}$ can be active in the same time as the process p (because y is coherent). The same reasoning apply when $\mathsf{in}(p) \cap \mathfrak{S} \neq \emptyset$.

Each story transition of the form $\mathfrak{s}_{\mathsf{e}} \xrightarrow{\mathtt{p}_1} \mathfrak{s}_{\mathtt{f}}$ has the following corresponding transitions in the general semantics: $\mathtt{f}_0 \xrightarrow{\mathtt{p}_1} \mathtt{f}_1$ if $e \neq \emptyset$ (production) and $\mathtt{e}_1 \xrightarrow{\{\mathtt{p}_1\} \cup \mathsf{done}^\star(p)} \mathtt{e}_0$ if $f \neq \emptyset$ (consumption).

Given any sequence of transitions in the stories semantics from y to y' (satisfying the above mentioned conditions), one can build a sequence of transitions in the general semantics by replacing all the stories transitions with the corresponding production transitions. Such a sequence of transitions leads to a state z of the general semantics with $\mathtt{p}_1 \in z$ and $\mathsf{done}^\star(p) \subseteq z$, that can be followed by all the consumption transitions, necessarily including all those corresponding to the stories transitions. $\square$

Property 2. *Let $x, x' \in S$ be states where no process is active. If $[\![x']\!]$ is reachable from $[\![x]\!]$ in the general semantics, then x' is* not necessarily *reachable from x in the stories semantics.*

Proof. Let us consider the following SBGN-PD map, with a unique story $\mathfrak{S} = \{A, A'\}$:

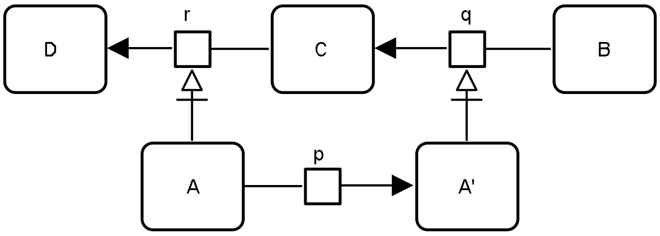

Let us consider the state x where only A and B are present and no process activated, and the state x' where only A' and D are present and no process activated:

$$x = \{\mathfrak{s}_{\mathtt{A}}, \mathrm{B}_1, \mathrm{C}_0, \mathrm{D}_0, \mathrm{p}_0, \mathrm{q}_0, \mathrm{r}_0\}$$
$$x' = \{\mathfrak{s}_{\mathtt{A}'}, \mathrm{B}_0, \mathrm{C}_0, \mathrm{D}_1, \mathrm{p}_0, \mathrm{q}_0, \mathrm{r}_0\}$$

The full transition graph from x with the stories semantics is reproduced below: the state x' is not connected to x, therefore, x' is not reachable from x with the stories semantics.

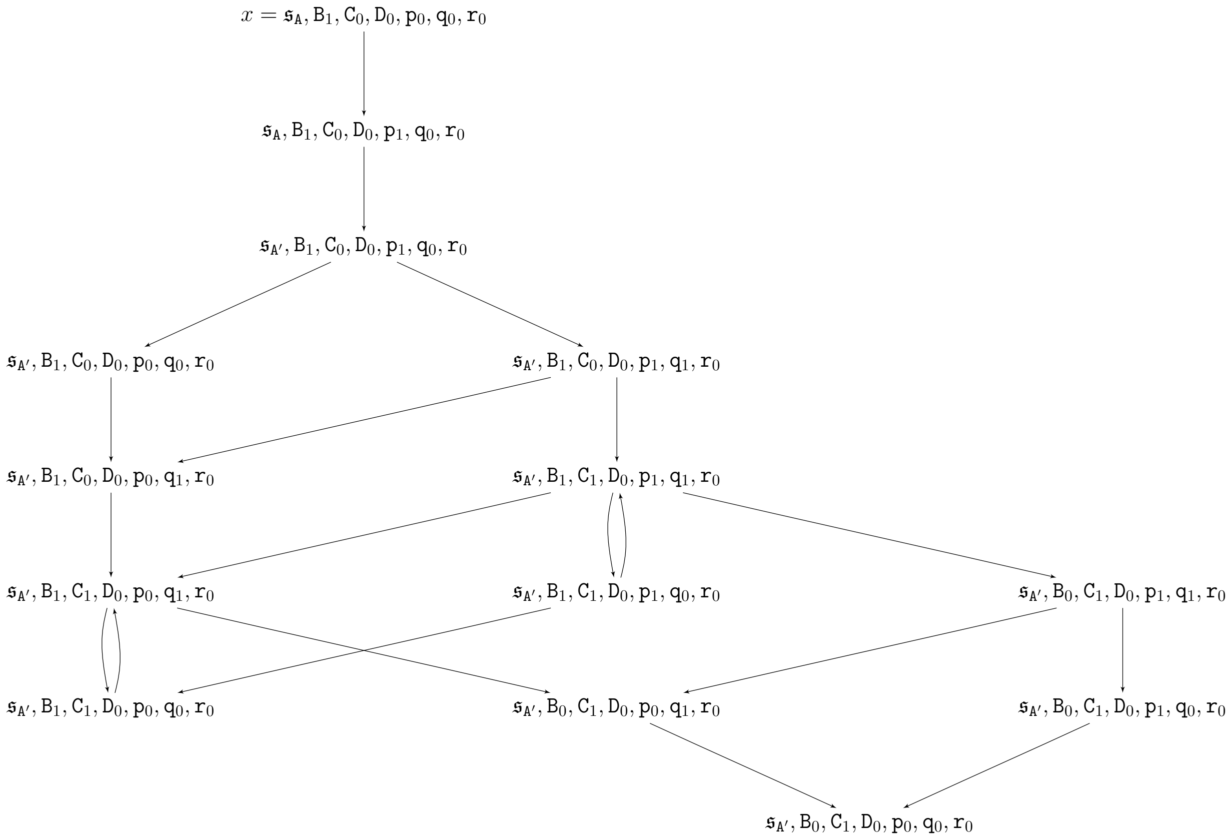

However, $[\![x']\!]$ is reachable from $[\![x]\!]$ in the general semantics by applying the following sequence of transitions:

$$\mathtt{p}_0 \xrightarrow{\mathtt{A}_1} \mathtt{p}_1, \mathtt{A'}_0 \xrightarrow{\mathtt{p}_1} \mathtt{A'}_1, \mathtt{q}_0 \xrightarrow{\mathtt{B}_1,\mathtt{A'}_1} \mathtt{q}_1, \mathtt{C}_0 \xrightarrow{\mathtt{q}_1} \mathtt{C}_1, \mathtt{r}_0 \xrightarrow{\mathtt{C}_1,\mathtt{A}_1} \mathtt{r}_1, \mathtt{D}_0 \xrightarrow{\mathtt{r}_1} \mathtt{D}_1,$$

$$\mathtt{A}_1 \xrightarrow{\mathtt{p}_1} \mathtt{A}_0, \mathtt{B}_1 \xrightarrow{\mathtt{q}_1} \mathtt{B}_0, \mathtt{C}_1 \xrightarrow{\mathtt{r}_1} \mathtt{C}_0, \mathtt{p}_1 \xrightarrow{\mathtt{A'}_1} \mathtt{p}_0, \mathtt{q}_1 \xrightarrow{\mathtt{C}_1} \mathtt{q}_0, \mathtt{r}_1 \xrightarrow{\mathtt{D}_1} \mathtt{r}_0$$

□

Property 3. *Given $x \in S$, if $[\![x]\!]$ is a fixed point in the general semantics, then x is a fixed point in the stories semantics.*

Proof sketch. We first remark that if $[\![x]\!]$ is a fixed point in the general semantics, then all the processes are inactive in $[\![x]\!]$. Indeed, for any process $p \in \mathcal{P}$, for each $e \in \mathsf{out}(p)$, $\mathtt{e}_1$ is always eventually reachable as long as $\mathtt{p}_1$ is present. Indeed the automaton $\mathtt{e}$ has only two states $0, 1$, and the transition $\mathtt{e}_0 \to \mathtt{e}_1$ is conditioned by $\mathtt{p}_1$ only. Therefore, the local states $\{\mathtt{p}_1\} \cup \mathsf{done}(p)$ are always eventually reachable from a state where $\mathtt{p}_1$ is present. Hence, the process p always eventually gets de-activated.

Finally, by lemma 1, if a process can get activated in x, it can get activated in $[\![x]\!]$. □

Property 4. *Given $x \in S$, if x is a fixed point in the stories semantics it does not imply that $[\![x]\!]$ is a fixed point in the general semantics.*

Proof. Let us consider the following SBGN-PD map, with the story $\mathfrak{S} = \{\emptyset, A, A'\}$.

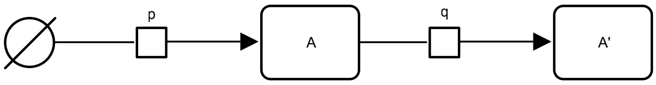

In the stories semantics, the state $x = \{\mathfrak{s}_{A'}, \mathtt{p}_0, \mathtt{q}_0\}$ is a fixed point. In the general semantics, the state $[\![x]\!] = \{\mathtt{A}_0, \mathtt{A'}_1, \mathtt{p}_0, \mathtt{q}_0\}$ is not a fixed point, as the transition $\mathtt{p}_0 \xrightarrow{\emptyset} \mathtt{p}_1$ can be executed. □
